# Ph_3_P-mediated decarboxylative ring-opening of maleic anhydride by thiolic compounds: formation of two carbon–sulfur bonds†

**DOI:** 10.1039/d3ra00294b

**Published:** 2023-03-20

**Authors:** N. Nowrouzi, M. Abbasi, Z. Zellifard

**Affiliations:** a Department of Chemistry, Faculty of Nano and Bio Science and Technology, Persian Gulf University Bushehr 75169 Iran nowrouzi@pgu.ac.ir +98-77-33441494 +98-77-31222341

## Abstract

In this study, a simple and efficient method for the formation of carbon–sulfur bonds is described. In this process, ring opening of maleic anhydride by thiols or disulfides and triphenylphosphine led to the formation of sulfide products *via* formation of two carbon-sulfur bonds.

## Abstract 2

Synthesis of *S*-aryl-3-(arylthio)propanethioate from the reaction of maleic anhydride and thiolic compounds in the presence of Ph_3_P.

## Graphical abstract

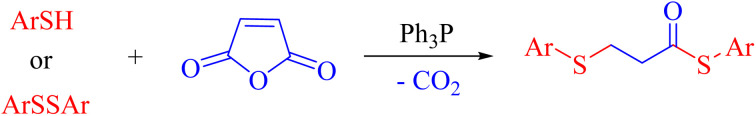

## 1 Introduction

Tertiary phosphines are widely used in organic synthesis due to their nucleophilic and reducing properties. They are also widely used as ligands in organometallic reactions or as catalysts in organic reactions.^1^ Reactions in which the tertiary phosphine acts as the catalyst are usually initiated by the nucleophilic addition of phosphine to activated carbon–carbon multiple bonds to form β-phosphonium α-carbanion species.^2^ For the first time, Horner isolated such zwitterionic species from the reaction of triethyl- and triphenylphosphine with ethylenemalononitrile.^2^ In 1968, Morita used a phosphonium-carbanion adduct to form α-hydroxymethyl acrylates.^3^ In 1972, Baylis and Hillman reported their Morita reaction catalyzed by an amine.^4^ In the same direction, Rauhut and Currier reported the first tributylphosphine-catalyzed dimerization of acrylates for the synthesis of α-methylene succinates.^5^ Contemporarily, Winterfeldt^6^ demonstrated the synthesis of butenolides from dimethyl acetylenedicarboxylate (DMAD) using a phosphine catalyst. Since then, nucleophilic phosphine catalysis has emerged as a powerful strategy for constructing important and useful structures and today, provides a reliable tool for the preparation of synthetic intermediates and products in all fields of organic chemistry, including pharmaceutical agents, natural products, ligands, and materials.^7,8^

Organic sulfurs are valuable intermediates for the synthesis of biologically active compounds and also in industry.^9^ For example, sulfur compounds are widely used in the manufacture of detergents, bleach, palms and dyes, wares as well as in the textile industry.^10^ Therefore, the formation of carbon–sulfur bond is very important and many efforts have been made in this field.^11^

## 2 Results and discussion

In view of the above remarks and in continuation of our work in this area,^12^ we decided to provide a simple and efficient method for the synthesis of sulfur compounds using thiols as the most available source of sulfur along with maleic anhydride, in the presence of catalytic amounts of triphenylphosphine.

**Scheme 1:**
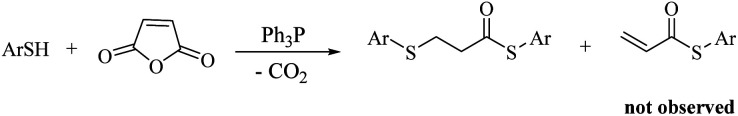
Carbon–sulfur bond formation catalyzed by triphenylphosphine.

At first, we chose a model reaction: addition of thiophenol (1.0 mmol) to maleic anhydride (1.0 mmol) in the presence of triphenylphosphine (0.1 mmol) in toluene under reflux conditions. Monitoring the progress of the reaction by thin-layer chromatography after 24 h, indicated formation of a product, but part of the maleic anhydride remained intact in the reaction medium. Spectroscopic identification of the product confirmed two molecules of thiol reacted with one of the maleic anhydride molecules, forming *S*-phenyl-3-(phenylthio)propanethioate as sole product (40%) (Scheme 1).

Then, attempts to improve the reaction yield by changing the ratio of reactants, solvent, temperature and amount of catalyst were made. The results of these studies are summarized in Table 1. Decreasing the amount of maleic anhydride up to 0.8 mmol did not alter the reaction yield, however further reduction, decreased the yield (Table 1, entries 2–4). Increasing the amount of triphenylphosphine produced satisfactory results (Table 1, entries 5–8). A significant increase of product yield was detected by increasing the catalyst up to 0.5 mmol (Table 1, entry 7). An increase beyond 0.5 mmol did not cause a noticeable change (Table 1, entry 8). Another control experiment confirmed that the reaction does not occur in the absence of triphenylphosphine (Table 1, entry 9). Inspired by the Baylis–Hillman reaction, we decided to use tertiary amines instead of triphenylphosphine and check the results. For this purpose, 0.5 mmol of Dabco or Bu_3_N were used instead of triphenylphosphine, neither of which had better or similar results (Table 1, entries 10 and 11). Since triphenylphosphine is easily converted to the corresponding oxide, in another attempt, triphenylphosphine oxide was prepared separately and used as a catalyst, in which case no product was formed (Table 1, entry 12). Thus triphenylphosphine, not its oxide, plays a catalytic role in these reactions. After determining the type and amount of catalyst, we further screened the reaction with various protic and aprotic solvents such as CH_3_CN, H_2_O, polyethylene glycol (PEG-200) and DMF (Table 1, entries 13–16). Among the solvents used, CH_3_CN showed similar results with toluene and in other solvents, the results were not satisfactory. So, CH_3_CN was used as the solvent in subsequent experiments. Finally, the reaction temperature was optimized. Decreasing the temperature, resulted the product in lower yield (Table 1, entries 17 and 18), therefore refluxing acetonitrile was found to be the optimal reaction conditions.

**Table 1:** Optimization of reaction conditions

Entry	Catalyst (mmol)	Solvent/temperature (^°^C)	Maleic anhydride	Yield^a^^,^^b^ (%)
1	Ph_3_P (0.1)	Toluene (reflux)	1.0	40
2	Ph_3_P (0.1)	Toluene (reflux)	0.8	38
3	Ph_3_P (0.1)	Toluene (reflux)	0.7	30
4	Ph_3_P (0.1)	Toluene (reflux)	0.6	24
5	Ph_3_P (0.2)	Toluene (reflux)	0.8	55
6	Ph_3_P (0.4)	Toluene (reflux)	0.8	73
7	Ph_3_P (0.5)	Toluene (reflux)	0.8	88
8	Ph_3_P (0.6)	Toluene (reflux)	0.8	90
9	—	Toluene (reflux)	0.8	—
10	Dabco (0.5)	Toluene (reflux)	0.8	—
11	Bu_3_N (0.5)	Toluene (reflux)	0.8	—
12	Ph_3_PO (0.5)	Toluene (reflux)	0.8	—
13	Ph_3_P (0.5)	CH_3_CN (reflux)	0.8	86
14	Ph_3_P (0.5)	H_2_O (100)	0.8	—
15	Ph_3_P (0.5)	PEG-200 (120)	0.8	20
16	Ph_3_P (0.5)	DMF (120)	0.8	15
17	Ph_3_P (0.5)	CH_3_CN (40)	0.8	54
18	Ph_3_P (0.5)	CH_3_CN (r. t.)	0.8	Trace

a Isolated yield.

b In all entries the reaction time is 24 hours.

The obtained optimum conditions for the reaction are as follows: thiol (1.0 mmol), maleic anhydride (0.8 mmol) in the presence of 0.5 mmol of triphenylphosphine in acetonitrile at reflux conditions.

After obtaining the optimal conditions, to develop the catalyst performance, different thiols possessing either electron-donating or electron-withdrawing substituents were examined with maleic anhydride in the presence of catalytic amount of triphenylphosphine, which afforded high yields of the corresponding sulfur products. The results are presented in Table 2.

**Table 2:** Synthesis of *S*-aryl-3-(arylthio)propanethioate from maleic anhydride and thiols catalyzed by triphenylphosphine^a^

Entry	ArSH	Product	Time (h)	Yield^b^ (%)
1	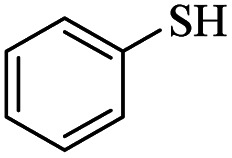	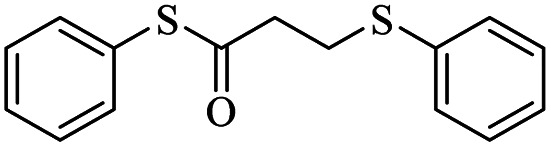	24	86
2	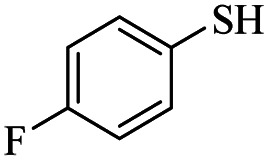	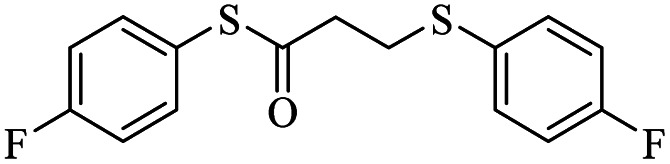	18	82
3	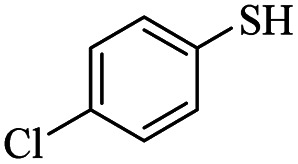	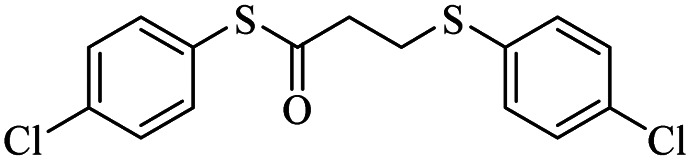	18	85
4	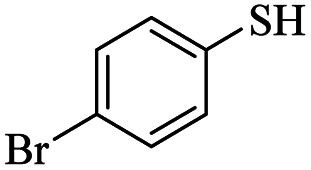	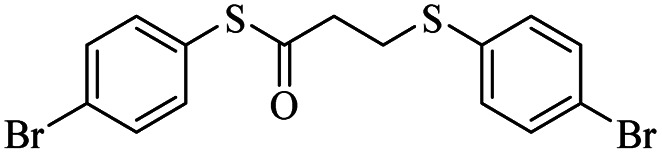	18	80
5	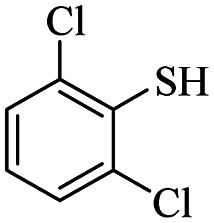	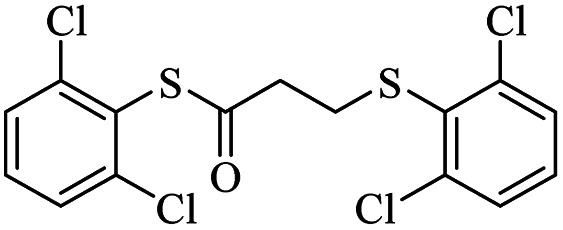	48	71
6	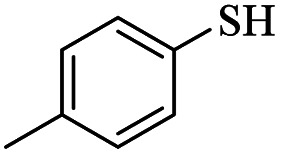	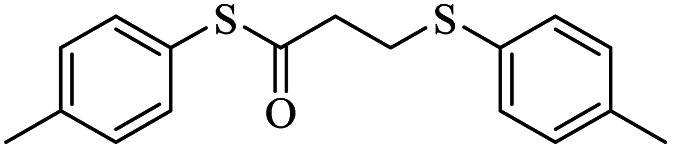	24	89
7	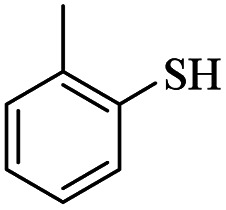	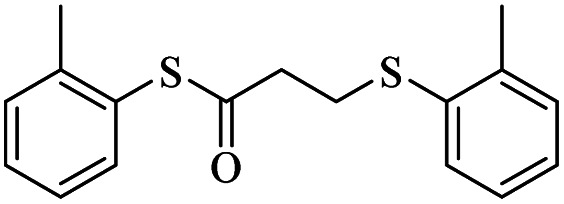	48	75
8	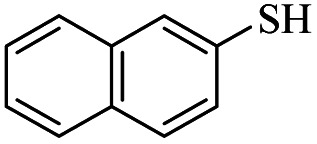	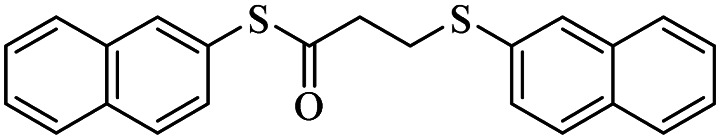	20	90
9	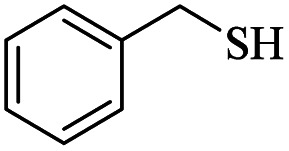	—	48	—
10	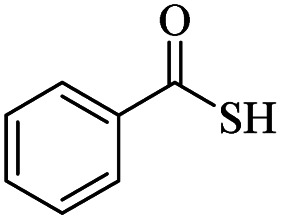	—	48	—

a Reaction conditions: thiol (1.0 mmol), maleic anhydride (0.8 mmol), triphenylphosphine (0.5 mmol) in acetonitrile at reflux.

b Isolated yields.

It would appear that the reaction is sensitive to steric effects to some extent, as 2,6-dichlorobenzenethiol and 2-methylbenzenethiol afforded the corresponding products in moderate yields, even in longer reaction time. Although aromatic thiols readily reacted with maleic anhydride in the present catalytic system and related products were produced, aliphatic thiol such as benzyl mercaptan did not produce any products. In another attempt, thiobenzoic acid replaced thiol. In this case, also, no product was formed even after 48 hours. Increasing the amount of catalyst, adding base to the reaction medium, and performing the reaction in refluxing toluene did not help the reaction.

**Scheme 2:**
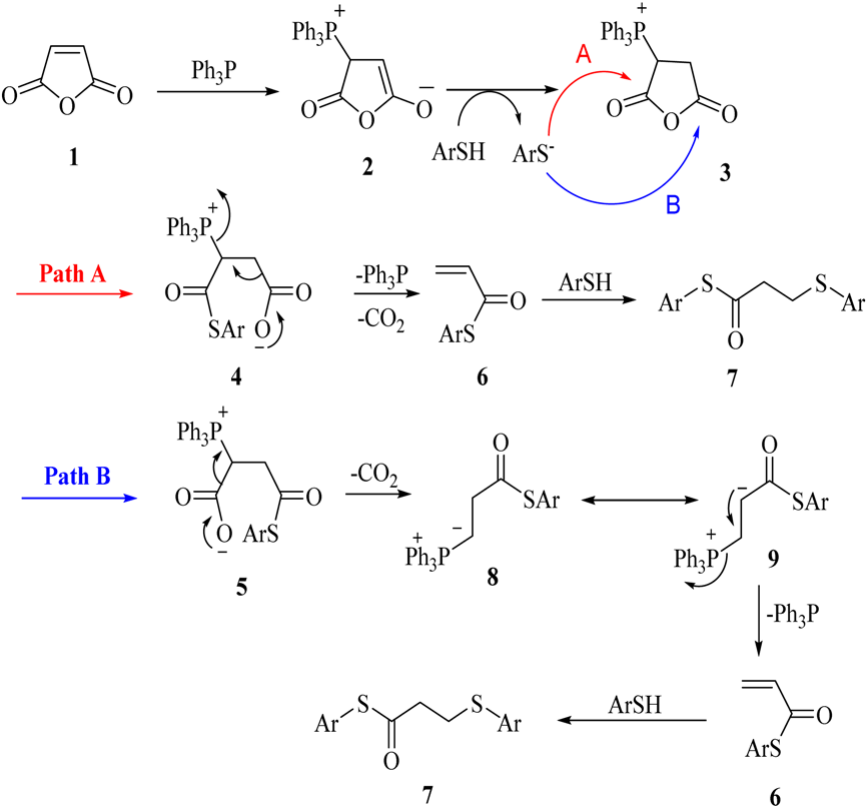
Carbon–sulfur bonds formation catalyzed by triphenylphosphine.

Two possible mechanisms for obtaining sulfide products are shown in Scheme 2. Phosphonium salt 2 is initially produced by the addition of triphenylphosphine to maleic anhydride 1. Protonation of 2 would lead to the phosphonium salt 3, which could undergo ring-opening by thiolate to provide phosphonium carboxylates 4 or 5*via* path A or path B. Decomposition of these carboxylates with the release of phosphine catalyst and carbon dioxide, produces acrylic thioester 6. Finally, the Michael addition of the second molecule of thiol on 6 produced the desired sulfide product 7.

After successfully ring-opening of malic anhydride with the help of thiols, we decided to use disulfide as a source of sulfur. In this case, by increasing the amount of triphenylphosphine in the reaction medium, thiolate anion can be created from disulfide to react with malic anhydride. For this purpose, diphenyl disulfide was prepared separately (1.0 mmol) and reacted with malic anhydride (0.8 mmol) in the presence of different amounts of triphenylphosphine under acetonitrile reflux conditions. The obtained results showed that the best amount of triphenylphosphine for this reaction was 3.3 mmol. Lower amounts decreased the yield and higher amounts did not further boost the yield above 90%. With these results, different aryl disulfides were synthesized and reacted with maleic anhydride according to Table 3 to form sulfide products.

**Table 3:** Synthesis of *S*-aryl-3-(arylthio)propanethioate from maleic anhydride and thiols catalyzed by triphenylphosphine^a^

Entry	ArSSAr	Product	Time (h)	Yield^b^ (%)
1	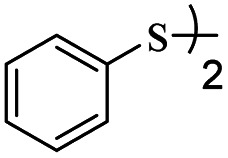	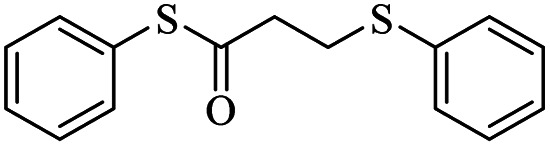	24	90
2	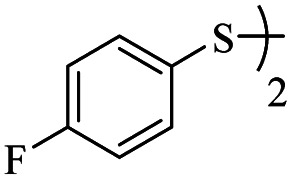	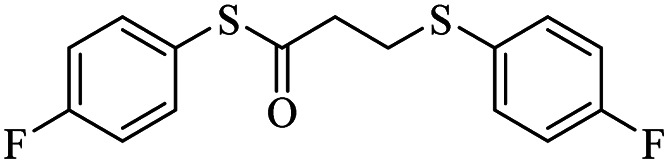	24	86
3	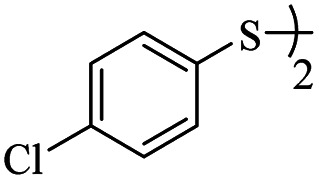	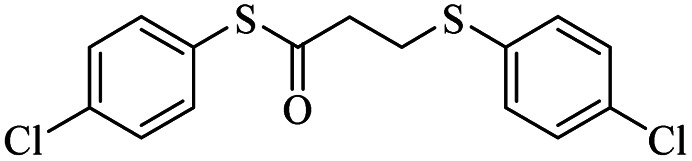	24	84
4	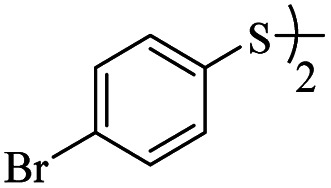	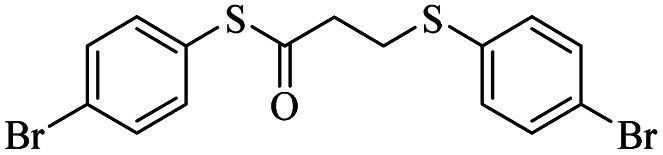	24	79
5	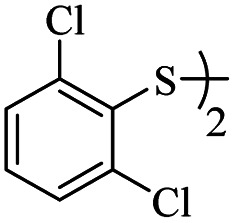	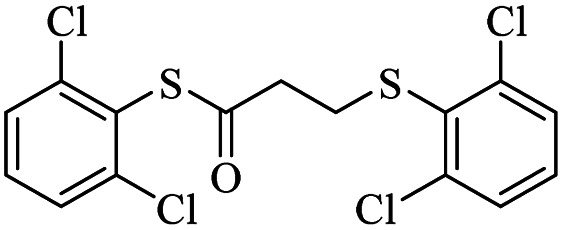	48	65
6	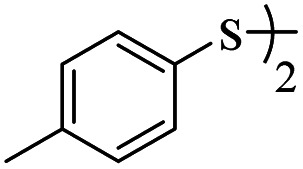	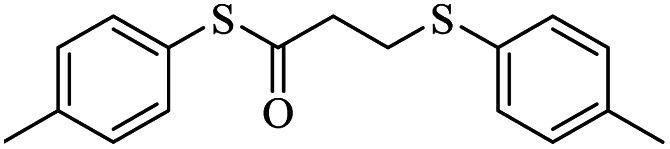	24	91
7	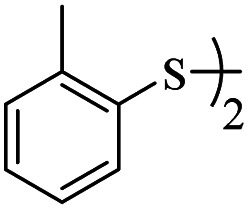	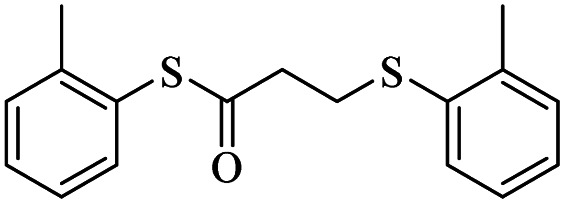	48	70
8	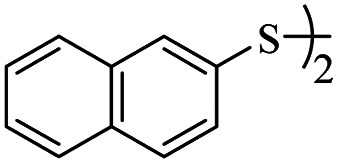	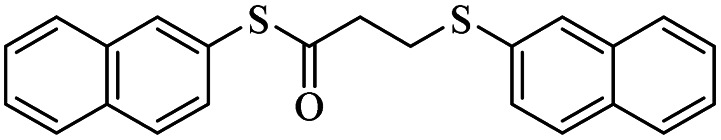	24	89
9	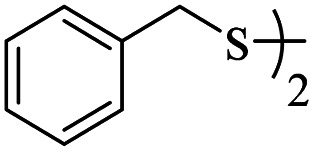	—	48	—

a Reaction conditions: disulfide (1.0 mmol), maleic anhydride (0.8 mmol), triphenylphosphine (3.3 mmol) in acetonitrile at reflux.

b Isolated yields.

The results show that different diaryl disulfides, like thiols, can be easily converted to the corresponding products in reaction with malic anhydride. As expected, the use of dialkyl disulfides in this direction did not lead to product formation (Table 3, entry 9).

**Scheme 3:**
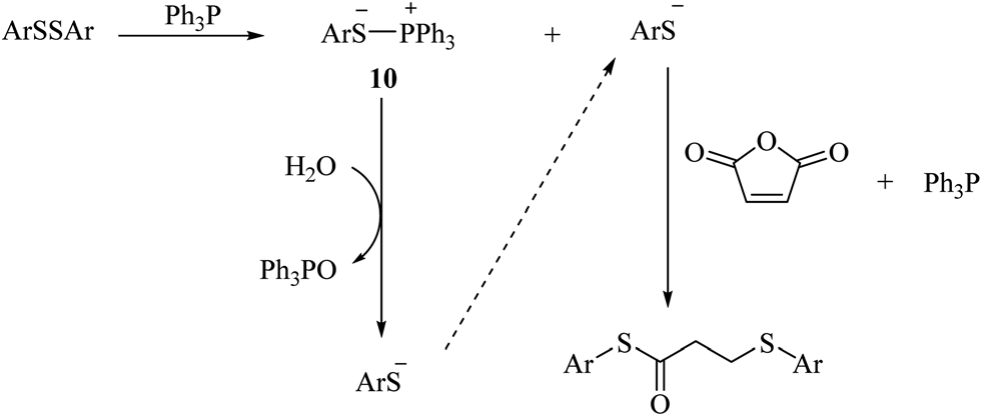
Proposed mechanism for the reaction of disulfide with maleic anhydride.

Diaryl disulfide is converted to thiolate anion and phosphonium salts 10 in the presence of triphenylphosphine. The thiolate anion reacts with malic anhydride according to the mechanism shown in Scheme 2 to produce the corresponding sulfur products. Phosphonium salt, on the other hand, reacts with a molecule of water to produce triphenylphosphine oxide together with the thiolate anion, which reacts again with maleic anhydride to form the product (Scheme 3).

## 3 Experimental

Chemicals were purchased from Merck and Aldrich chemical companies. The products were characterized by comparison of their spectral and physical data with those reported in the literature. For the recorded ^1^H NMR spectra we used Bruker (300 and 400 MHZ) Avance Ultrashield in pure deuterated CDCl_3_ solvents with tetramethylsilane (TMS) as internal standards. The reaction monitoring was accomplished by TLC on silica gel PolyGram SILG/UV254 plates. Column chromatography was carried out on columns of silica gel 60 (70–230 mesh).

### 3.1 General procedure for the synthesis of *S*-aryl-3-(arylthio)propanethioate from maleic anhydride and thiols

Thiol (1.0 mmol), maleic anhydride (0.8 mmol), and triphenylphosphine (0.5 mmol) were added to a flask containing 1 mL of acetonitrile. The mixture was heated in an oil bath at reflux with stirring and the reaction was followed by TLC analysis. After completion of the reaction, the solution was let to cool down to room temperature. The solvent was removed and the crude product was purified by chromatography over silica gel using *n*-hexane/ethyl acetate (10 : 1) as eluent to afford the pure product (Table 2).

### 3.2 General procedure for the synthesis of *S*-aryl-3-(arylthio)propanethioate from maleic anhydride and disulfides

To a mixture of disulfide (1.0 mmol) and maleic anhydride (0.8 mmol) in acetonitrile (1 mL), triphenylphosphine (3.3 mmol) was added and refluxed. After completion of the reaction (monitored by TLC), the mixture was cooled to room temperature. Then, the crude product was purified by silica gel column chromatography employing *n*-hexane/ethyl acetate (10 : 1) as the eluent to afford the pure product (Table 3).

### 3.3 Spectral data

#### 3.3.1 *S*-Phenyl-3-(phenylthio)propanethioate

Yellow oil, 0.236 g, 86% yield. ^1^H-NMR (400 MHz, CDCl_3_), *δ*(ppm): 7.44–7.42 (m, 7H, Ar), 7.36 (t, *J* = 7.2 Hz, 2H, Ar), 7.27 (t, *J* = 7.2 Hz, 1H, Ar), 3.27 (t, *J* = 7.6 Hz, 2H, CH_2_), 2.99 (t, *J* = 7.6 Hz, 2H, CH_2_). ^13^C-NMR (100 MHz, CDCl_3_), *δ*(ppm): 195.7, 134.9, 134.5, 130.2, 129.6, 129.2, 129.1, 127.2, 126.7, 43.2, 29.2. Anal. calcd for C_15_H_14_OS_2_: C, 65.66; H, 5.14; S, 23.37. Found: C, 65.79; H, 5.21; S, 23.25 (Table 2, entry 1).^13^

#### 3.3.2 *S*-(4-Fluorophenyl)-3-((4-fluorophenyl)thio)propanethioate

Yellow oil, 0.254 g, 82% yield. ^1^H-NMR (300 MHz, CDCl_3_), *δ*(ppm): 7.47–7.37 (m, 4H, Ar), 7.17–7.03 (m, 4H, Ar), 3.20 (dt, *J*_1_ = 6, *J*_2_ = 2.1 Hz, 2H, CH_2_), 2.94 (dt, *J*_1_ = 7.3, *J*_2_ = 1.8 Hz, 2H, CH_2_). ^13^C-NMR (75 MHz, CDCl_3_), *δ*(ppm): 195.5, 164.6 (d, *J* = 98.7 Hz), 161.3 (d, *J* = 95.8 Hz), 136.5 (d, *J* = 8.5 Hz), 133.5 (d, *J* = 8.1 Hz), 129.7, 122.5, 116.6 (d, *J* = 19.4 Hz), 116.3 (d, *J* = 19.3 Hz), 40.9, 29.6. Anal. calcd for C_15_H_12_F_2_OS_2_: C, 58.05; H, 3.90; S, 20.66. Found: C, 58.14; H, 3.80; S, 20.77 (Table 2, entry 2).^13^

#### 3.3.3 *S*-(4-Chlorophenyl)-3-((4-chlorophenyl)thio)propanethioate

Colorless oil, 0.291 g, 85% yield. ^1^H-NMR (400 MHz, CDCl_3_), *δ*(ppm): 7.56–7.54 (m, 2H, Ar), 7.50–7.41 (m, 6H, Ar), 3.34 (t, *J* = 4.8, 2H, CH_2_), 3.03 (t, *J* = 4.8, 2H, CH_2_). ^13^C-NMR (100 MHz, CDCl_3_), *δ*(ppm): 195.9, 135.1, 134.3, 134.2, 132.7, 131.7, 130.1, 129.5, 129.1, 40.3, 30.2. Anal. calcd for C_15_H_12_Cl_2_OS_2_: C, 52.48; H, 3.52; S, 18.68. Found: C, 52.51; H, 3.43; S, 18.55 (Table 2, entry 3).^13^

#### 3.3.4 *S*-(4-Bromophenyl)-3-((4-bromophenyl)thio)propanethioate

White solid, 0.346 g, 80% yield. ^1^H-NMR (400 MHz, CDCl_3_), *δ*(ppm): 7.57 (d, *J* = 8.4 Hz, 2H, Ar), 7.47 (d, *J* = 8.4 Hz, 2H, Ar), 7.27 (dd, *J*_1_ = 8.4, *J*_2_ = 2.8 Hz, 4H, Ar), 3.23 (t, *J* = 7.2 Hz, 2H, CH_2_), 2.97 (t, *J* = 7.2 Hz, 2H, CH_2_). ^13^C-NMR (100 MHz, CDCl_3_), *δ*(ppm): 194.8, 135.9, 134.1, 132.5, 131.8, 129.4, 126.2, 124.3, 120.8, 43.1, 29.2. Anal. calcd for C_15_H_12_Br_2_OS_2_: C, 41.69; H, 2.80; S, 14.84. Found: C, 41.56; H, 2.91; S, 14.80 (Table 2, entry 4).^13^

#### 3.3.5 *S*-(2,6-Dichlorophenyl)-3-((2,6-dichlorophenyl)thio)propanethioate

White solid, 0.292 g, 71% yield. ^1^H-NMR (400 MHz, CDCl_3_), *δ*(ppm): 7.47–7.43 (m, 4H, Ar), 7.35–7.23 (m, 2H, Ar), 3.27 (t, *J* = 8.0 Hz, 2H, CH_2_), 3.00 (t, *J* = 7.8 Hz, 2H, CH_2_). ^13^C-NMR (100 MHz, CDCl_3_), *δ*(ppm): 191.8, 141.7, 140.9, 131.7, 131.6, 130.4, 128.8, 128.7, 126.9, 43.7, 30.0. Anal. calcd for C_15_H_10_Cl_4_OS_2_: C, 43.71; H, 2.45; S, 15.56. Found C, 43.62; H, 2.55; S, 15.44 (Table 2, entry 5).

#### 3.3.6 *S*-(*p*-Tolyl)-3-(*p*-tolylthio)propanethioate

Yellow oil, 0.269 g, 89% yield. ^1^H-NMR (400 MHz, CDCl_3_), *δ*(ppm): 7.39–7.37 (m, 2H, Ar), 7.28–7.26 (m, 2H, Ar), 7.23–7.20 (m, 2H, Ar), 7.11–7.09 (m, 2H, Ar), 3.24 (t, *J* = 4.8 Hz, 2H, CH_2_), 2.98 (t, *J* = 4.8 Hz, 2H, CH_2_), 2.35 (broad, 6H, CH_3_). ^13^C-NMR (100 MHz, CDCl_3_), *δ*(ppm): 196.2, 138.9, 136.2, 134.2, 133.5, 130.6, 130.4, 129.9, 129.8, 40.6, 30.6, 21.45, 21.44. Anal. calcd for C_17_H_18_OS_2_: C, 67.51; H, 6.00; S, 21.20. Found: C, 67.44; H, 6.09; S, 21.33 (Table 2, entry 6).^13^

#### 3.3.7 *S-o*-Tolyl-3-(*o*-tolylthio)propanethioate

Yellow oil, 0.226 g, 75% yield. ^1^H-NMR (300 MHz, CDCl_3_), *δ*(ppm): 7.44–7.33 (m, 4H, Ar), 7.29–7.16 (m, 4H, Ar), 3.26 (t, *J* = 7.6 Hz, 2H, CH_2_), 3.00 (t, *J* = 7.6 Hz, 2H, CH_2_), 2.45 (s, 3H, CH_3_), 2.39 (s, 3H, CH_3_). ^13^C-NMR (75 MHz, CDCl_3_), *δ*(ppm): 195.3, 142.0, 138.5, 135.9, 134.2, 130.8, 130.4, 130.2, 129.2, 126.8, 126.67, 126.61, 126.5, 43.0, 28.5, 20.8, 20.5. Anal. calcd for C_17_H_18_OS_2_: C, 67.51; H, 6.00; S, 21.20. Found: C, 67.65; H, 6.01; S, 21.09 (Table 2, entry 7).

#### 3.3.8 *S*-Naphthalen-2-yl-3-(naphthalen-2-ylthio)propanethioate

White solid, 0.336 g, 90% yield. ^1^H-NMR (300 MHz, CDCl_3_), *δ*(ppm): 7.93 (t, *J* = 1.0 Hz, 1H, Ar), 7.90–7.80 (m, 7H, Ar), 7.58–7.48 (m, 5H, Ar), 7.44 (dd, *J*_1_ = 8.5, *J*_2_ = 1.8 Hz, 1H, Ar), 3.39 (t, *J* = 7.6 Hz, 2H, CH_2_), 3.08 (t, *J* = 7.6 Hz, 2H, CH_2_). ^13^C-NMR (75 MHz, CDCl_3_), *δ*(ppm): 196.0, 134.4, 133.7, 133.5, 133.4, 132.3, 132.1, 130.8, 128.8, 128.7, 128.4, 128.0, 127.9, 127.8, 127.7, 127.28, 127.26, 126.7, 126.6, 126.0, 124.5, 43.3, 29.1. Anal. calcd for C_23_H_18_OS_2_: C, 73.76; H, 4.84; S, 17.12. Found: C, 73.88; H, 4.90; S, 17.01 (Table 2, entry 8).^13^

## 4 Conclusions

In this study, ring opening of maleic anhydride by thiols catalyzed by triphenylphosphine led to the formation of two carbon-sulfur bonds. The first carbon sulfide bond was formed by the nucleophilic attack of thiolate anion on maleic anhydride, and the second bond was formed by Michael addition on *in situ* generated acrylic thioester. Various aromatic thiols were used and the desired products were produced in good to excellent yields by a simple method and using a cheap catalyst. Then, disulfide was used as a source of sulfur instead of thiol. In this case, using a larger amount of triphenylphosphine and producing thiolate in the reaction medium, the desired sulfide products were well formed by the presented mechanism. Aromatic disulfides with electron-donating and electron-withdrawing groups on the ring reacted well with maleic anhydride and the products were synthesized with acceptable yield.

## Conflicts of interest

There are no conflicts to declare.

## Supplementary Material

RA-013-D3RA00294B-s001

RA-013-D3RA00294B-s002
