# Supplementary material for: Ph3P-mediated decarboxylative ring-opening of maleic anhydride by thiolic compounds: formation of two carbon–sulfur bonds
Source: RSC Adv. 2023 Mar 20;13(14):9242–6. doi: 10.1039/d3ra00294b (PMC10026555; doi:10.1039/d3ra00294b)
Supplement: RA-013-D3RA00294B-s002 [file RA-013-D3RA00294B-s002.pdf]

Electronic Supplementary Material (ESI) for RSC Advances.
This journal is © The Royal Society of Chemistry 2023

Supporting Information

Ph_3P-mediated decarboxylative ring-opening of maleic anhydride by thiolic compounds: Formation of two carbon-sulfur bonds

Najmeh Nowrouzi,*[a] Mohammad Abbasi [a] and Zeinab Zellifard [a]

[a] Department of Chemistry, Faculty of Nano and Bio Science and Technology, Persian Gulf University, Bushehr 75169 Iran Address here

*E-mail address: nowrouzi@pgu.ac.ir.

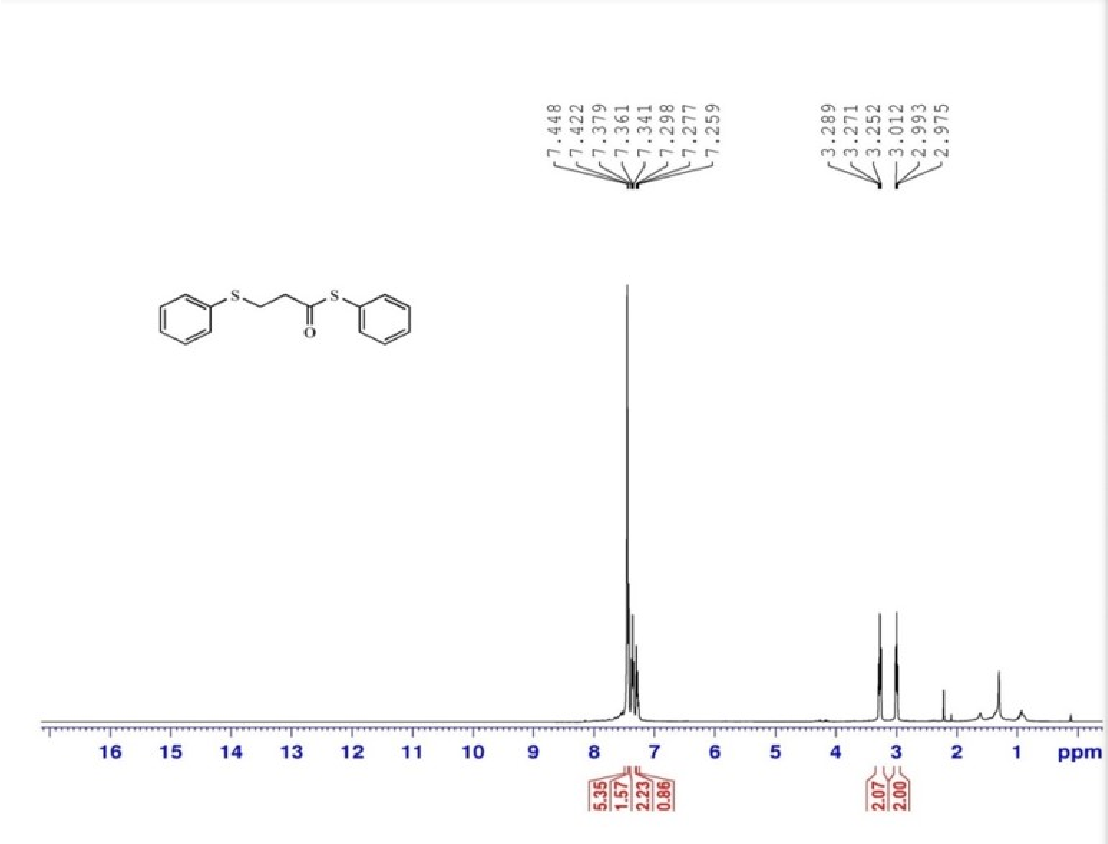
7.448
7.422
7.379
7.361
7.341
7.298
7.277
7.259
3.289
3.271
3.252
3.012
2.993
2.975
S
S
O
16 15 14 13 12 11 10 9 8 7 6 5 4 3 2 1 ppm
5.35
1.57
2.23
0.86
2.07
2.00

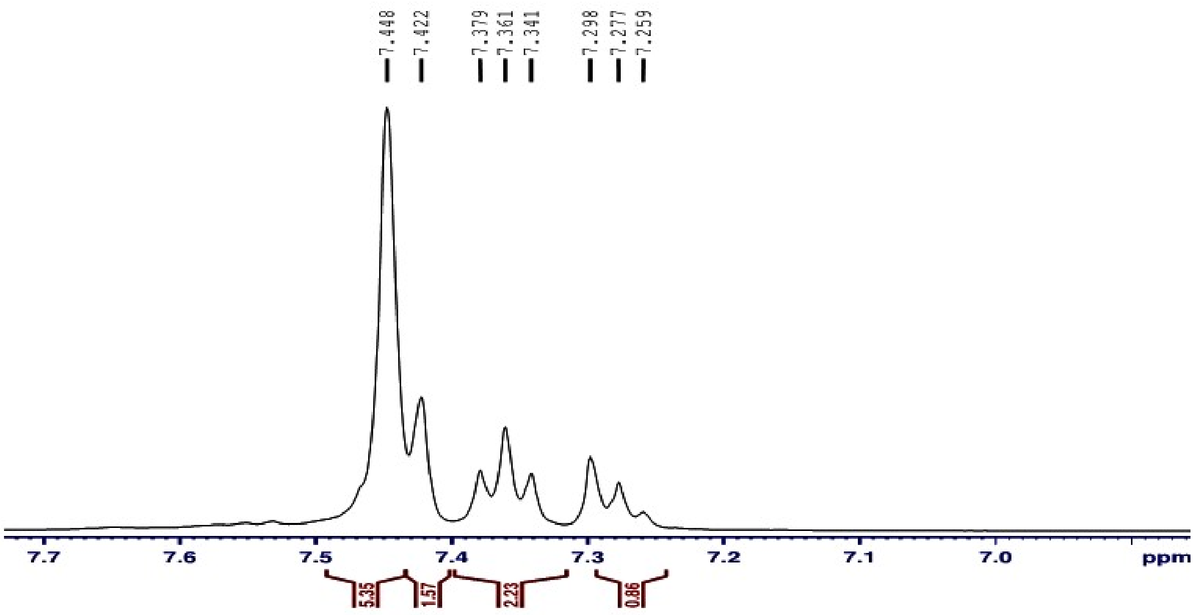
7.448
7.422
7.379
7.361
7.341
7.298
7.277
7.259
7.7 7.6 7.5 7.4 7.3 7.2 7.1 7.0 ppm
5.35
1.57
2.23
0.86

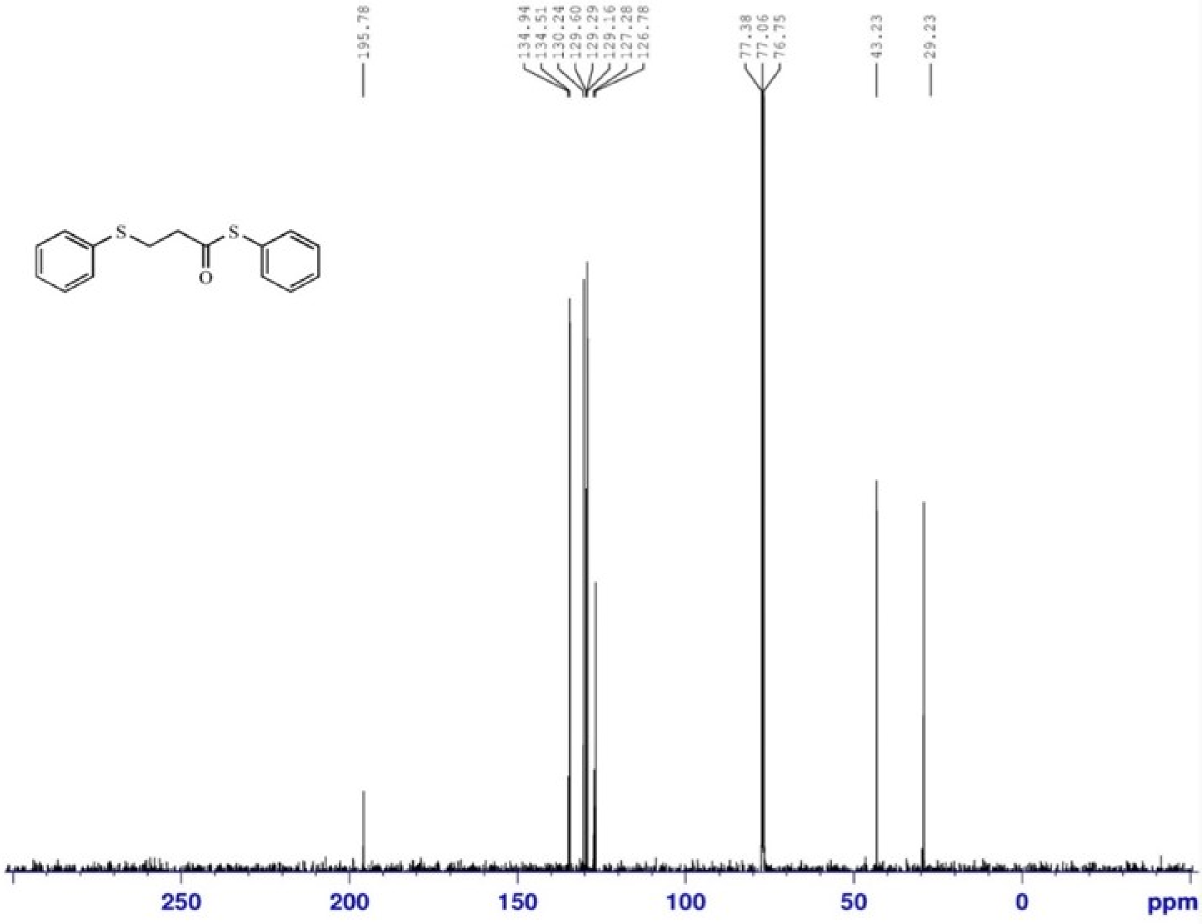
195.78
134.94
134.51
130.24
129.60
129.29
129.16
127.28
126.78
77.38
77.06
76.75
43.23
29.23
S
S
O
250
200
150
100
50
0
ppm

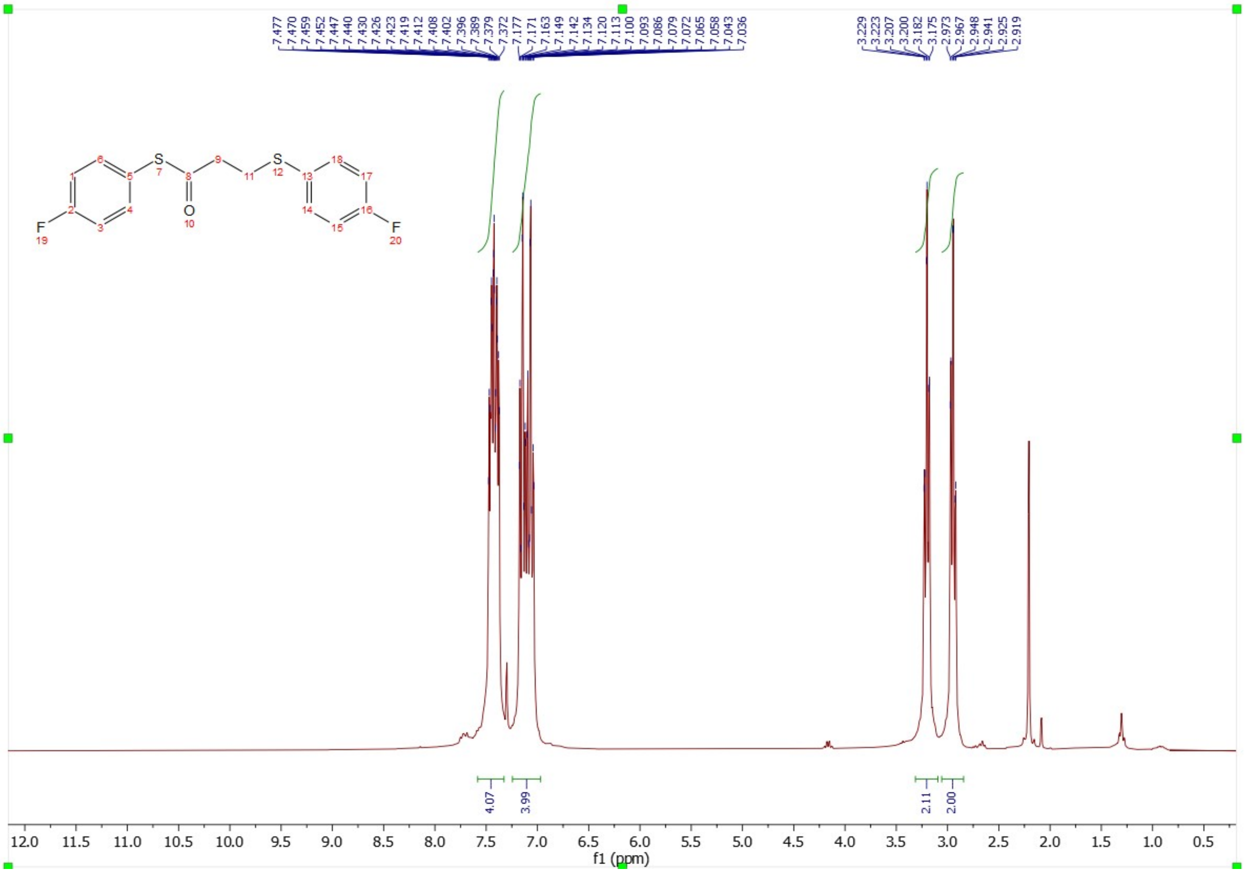
7.477 7.470 7.459 7.452 7.447 7.440 7.430 7.426 7.423 7.419 7.412 7.408 7.402 7.396 7.389 7.379 7.372 7.177 7.171 7.163 7.149 7.142 7.134 7.120 7.113 7.100 7.093 7.086 7.079 7.072 7.065 7.058 7.043 7.036
3.229 3.223 3.207 3.200 3.182 3.175 2.973 2.967 2.948 2.941 2.925 2.919
4.07
3.99
2.11
2.00
f1 (ppm)

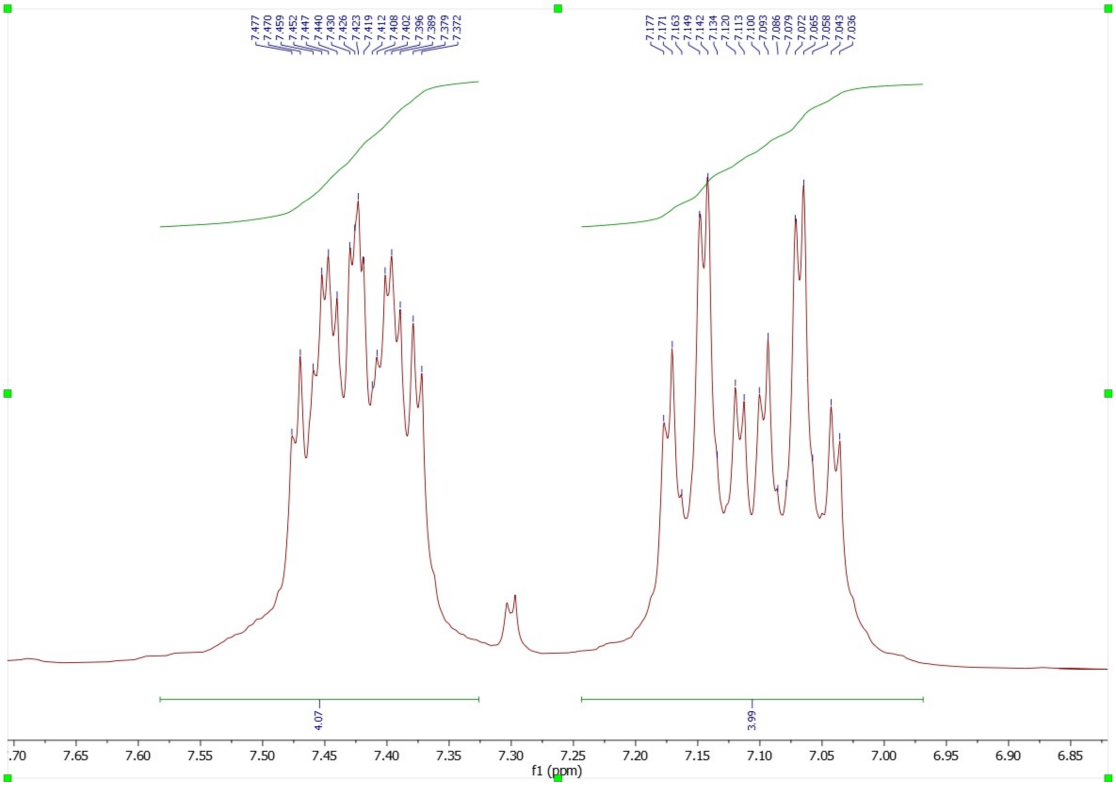
7.477 7.470 7.459 7.452 7.447 7.440 7.430 7.426 7.423 7.419 7.412 7.408 7.402 7.396 7.389 7.379 7.372
7.177 7.171 7.163 7.149 7.142 7.134 7.120 7.113 7.100 7.093 7.086 7.079 7.072 7.065 7.058 7.043 7.036
4.07
3.99
f1 (ppm)

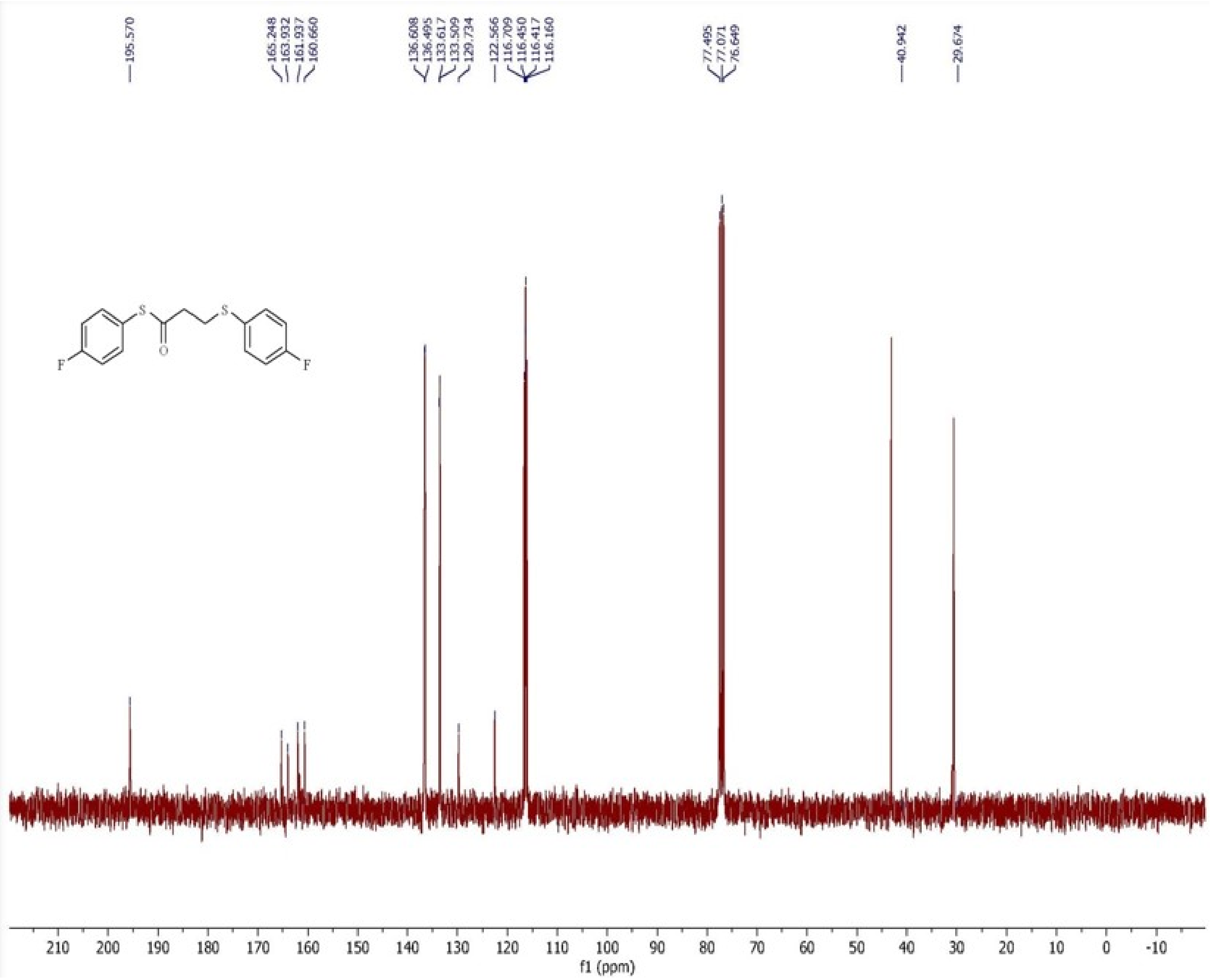
195.570
165.248
163.932
161.937
160.660
136.608
136.495
133.617
133.509
129.734
122.566
116.709
116.450
116.417
116.160
77.495
77.071
76.649
40.942
29.674
F
S
O
S
F
210 200 190 180 170 160 150 140 130 120 110 100 90 80 70 60 50 40 30 20 10 0 -10
f1 (ppm)

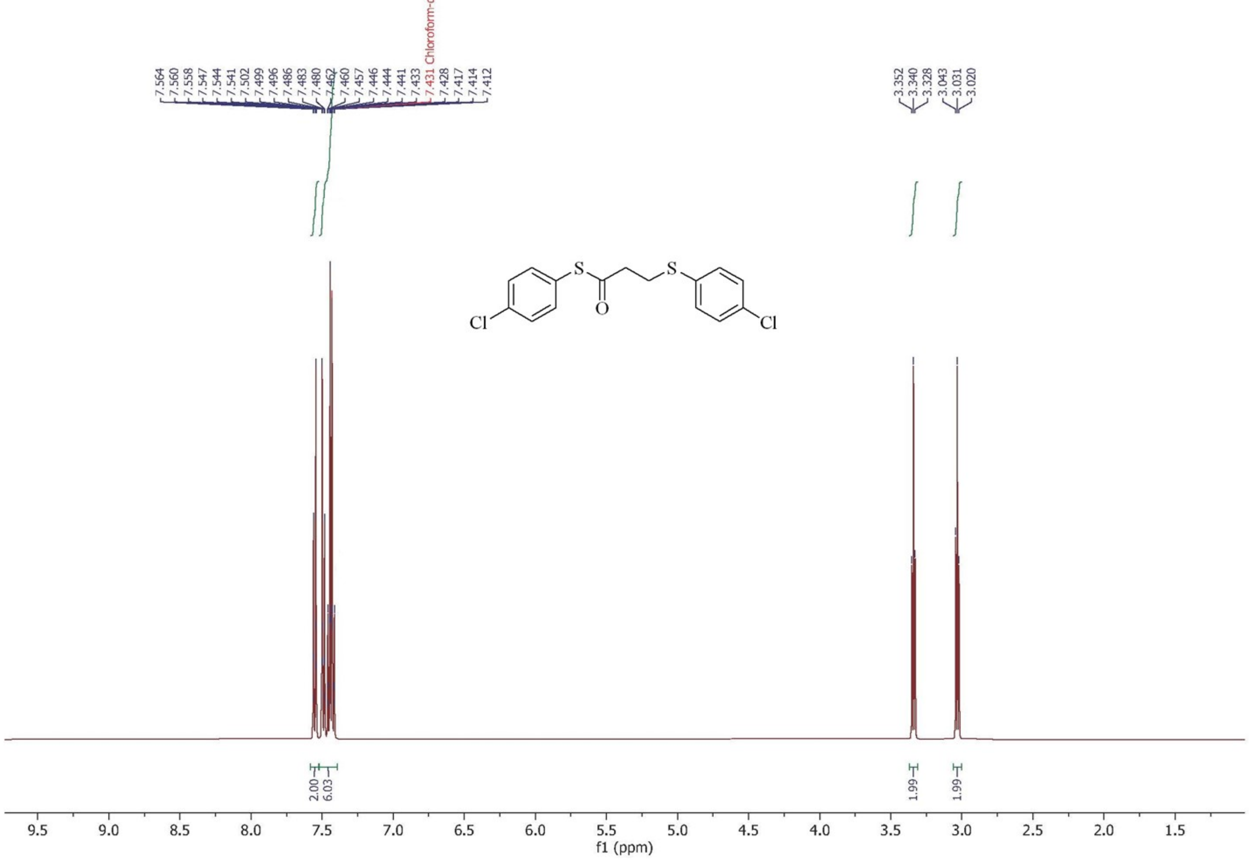

7.564 7.560 7.558 7.547 7.544 7.541 7.502 7.499 7.496 7.486 7.483 7.480 7.462 7.460 7.457 7.446 7.444 7.441 7.433 7.431 Chloroform-d 7.428 7.417 7.414 7.412
3.352 3.340 3.328 3.043 3.031 3.020
Cl
S
O
S
Cl
2.00 6.03 1.99 1.99
f1 (ppm)

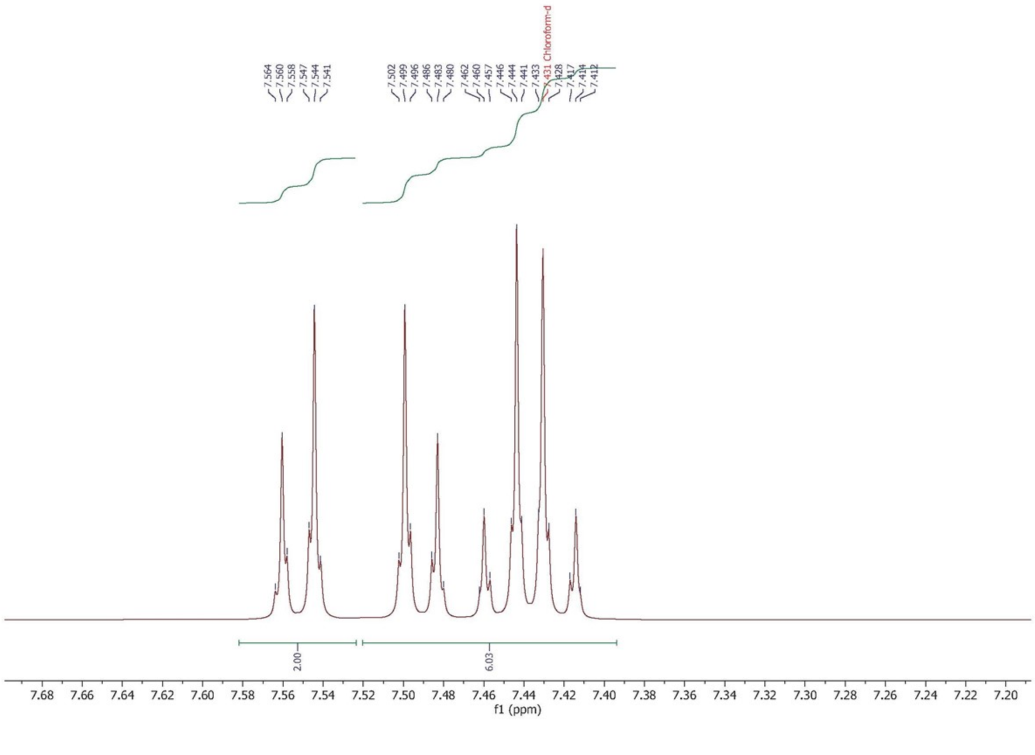

7.564 7.560 7.558 7.547 7.544 7.541 7.502 7.499 7.496 7.486 7.483 7.480 7.462 7.460 7.457 7.446 7.444 7.441 7.433 7.431 Chloroform-d 7.428 7.417 7.414 7.412
2.00 6.03
f1 (ppm)

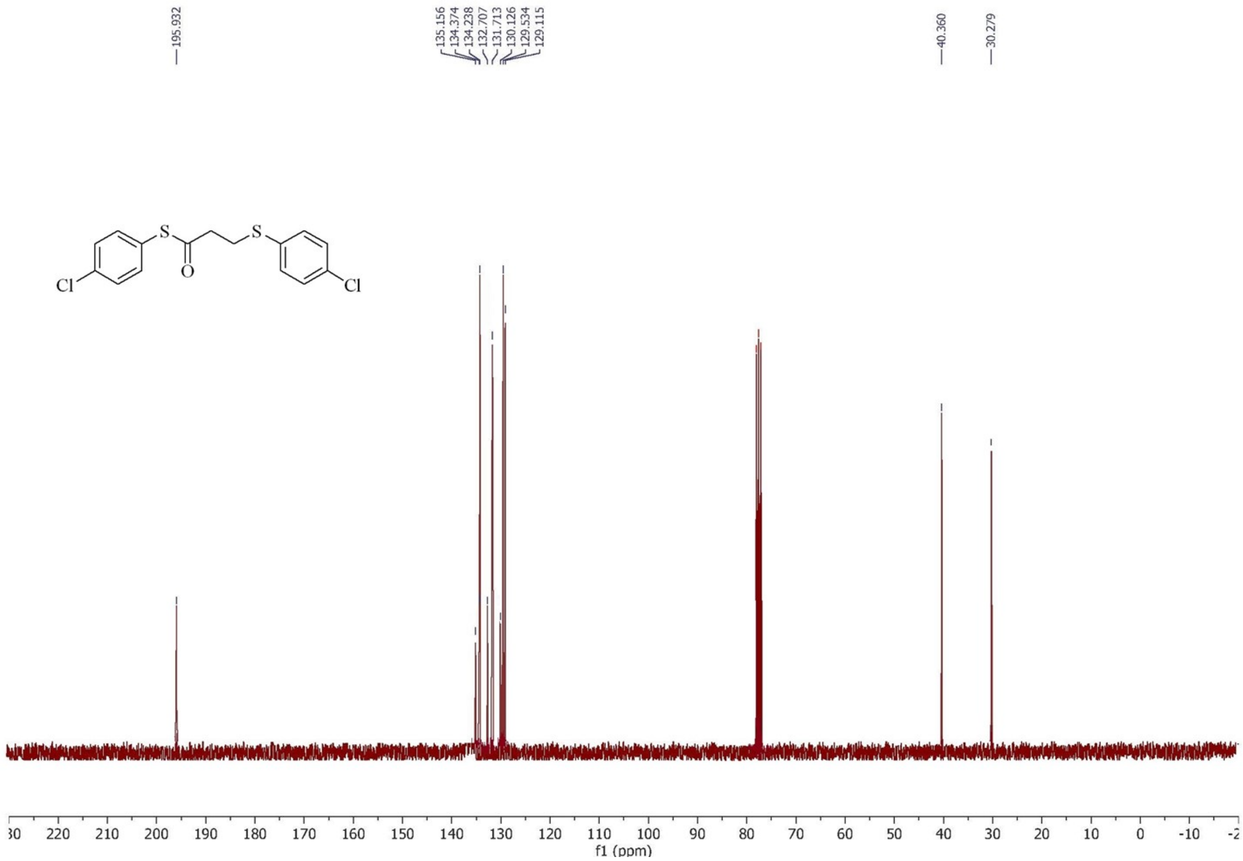

195.932
135.156
134.374
134.238
132.707
131.713
130.126
129.534
129.115
40.360
30.279
S
O
S
Cl
Cl
30
220
210
200
190
180
170
160
150
140
130
120
110
100
90
80
70
60
50
40
30
20
10
0
-10
f1 (ppm)

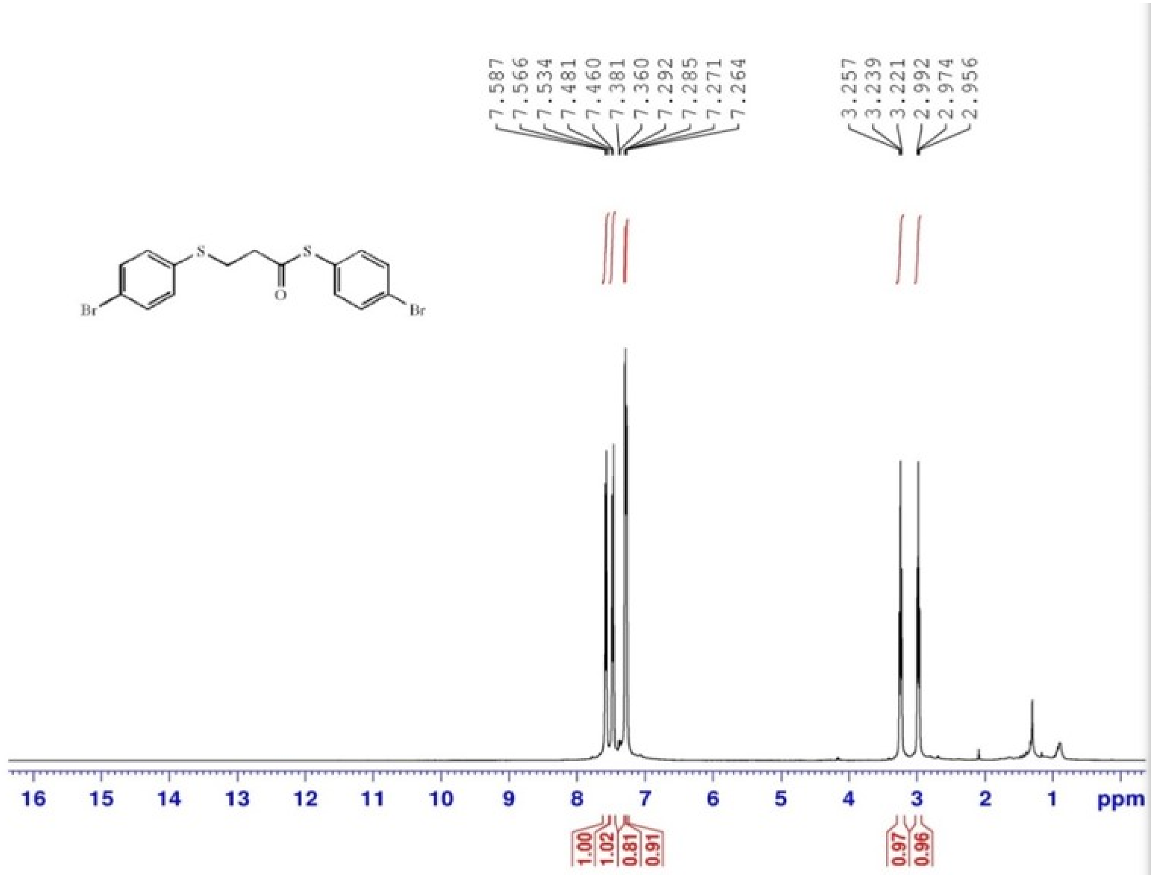

7.587
7.566
7.534
7.481
7.460
7.381
7.360
7.292
7.285
7.271
7.264
3.257
3.239
3.221
2.992
2.974
2.956
Br
S
O
S
Br
16 15 14 13 12 11 10 9 8 7 6 5 4 3 2 1 ppm
1.00
1.02
0.81
0.91
0.97
0.96

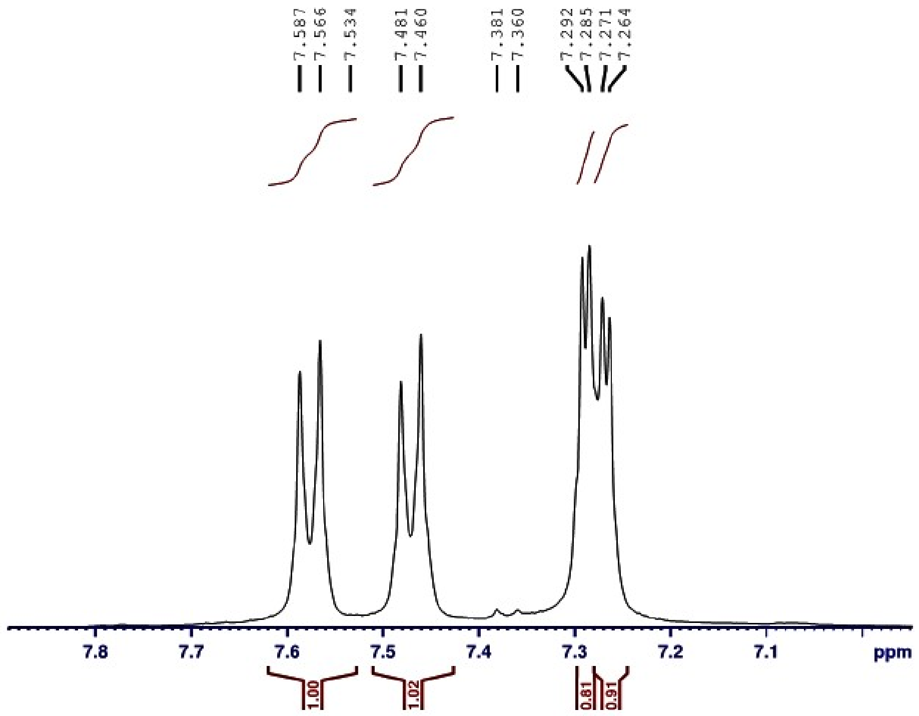

7.587
7.566
7.534
7.481
7.460
7.381
7.360
7.292
7.285
7.271
7.264
7.8 7.7 7.6 7.5 7.4 7.3 7.2 7.1 ppm
1.00
1.02
0.81
0.91

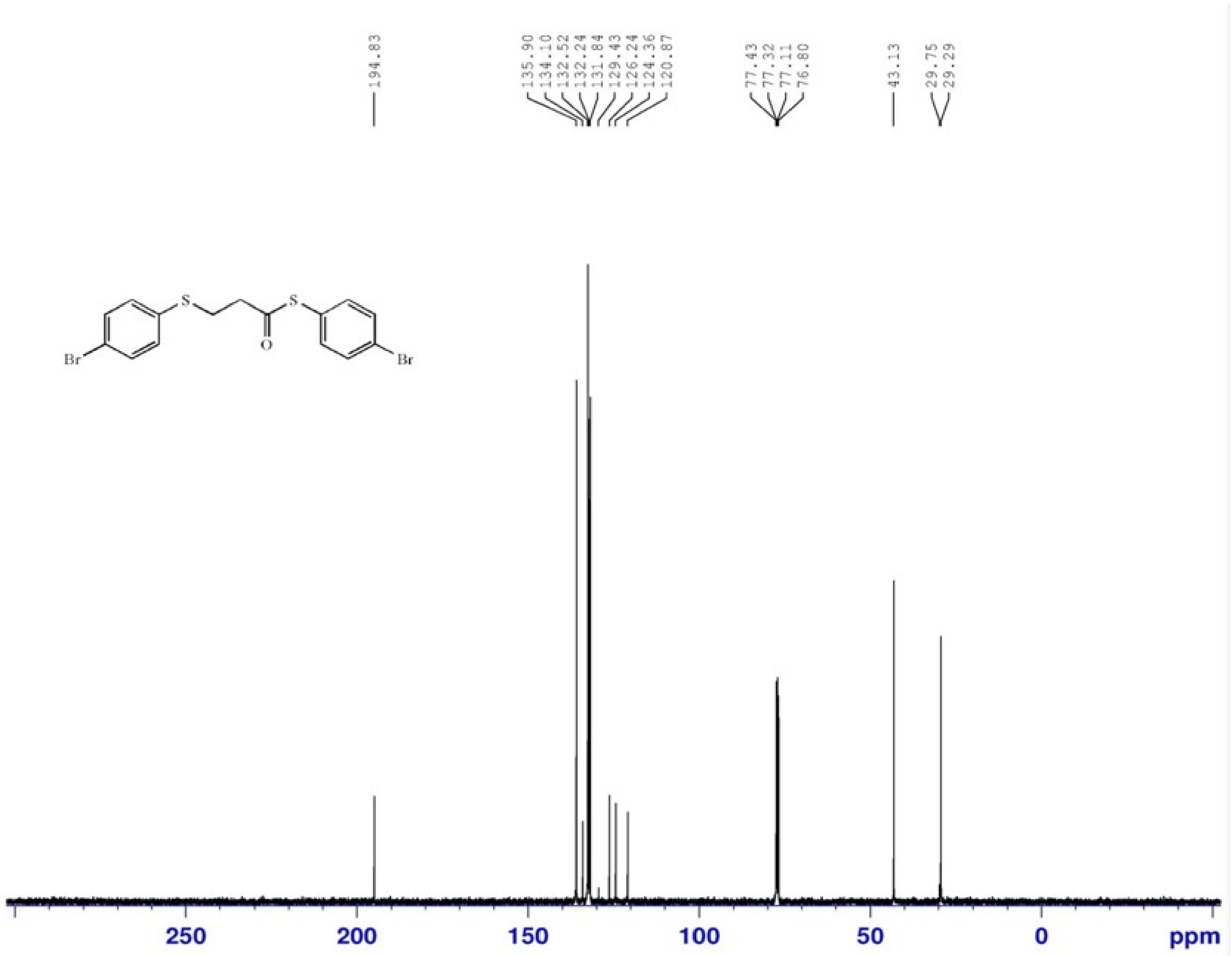
194.83
135.90
134.10
132.52
132.24
131.84
129.43
126.24
124.36
120.87
77.43
77.32
77.11
76.80
43.13
29.75
29.29
S
S
O
Br
Br
250
200
150
100
50
0
ppm

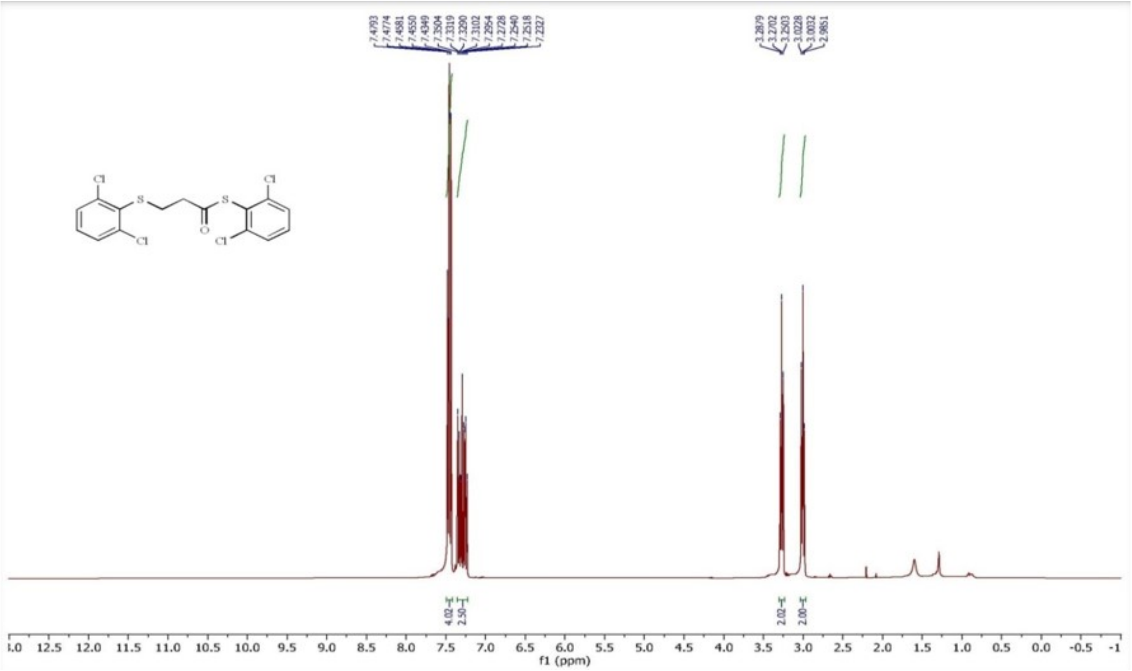
7.4793
7.4774
7.4581
7.4550
7.4349
7.3304
7.3319
7.3290
7.3102
7.2954
7.2728
7.2540
7.2518
7.2327
3.2879
3.2702
3.2503
3.0228
3.0032
2.9851
Cl
S
S
O
4.02
2.50
2.02
2.00
f1 (ppm)

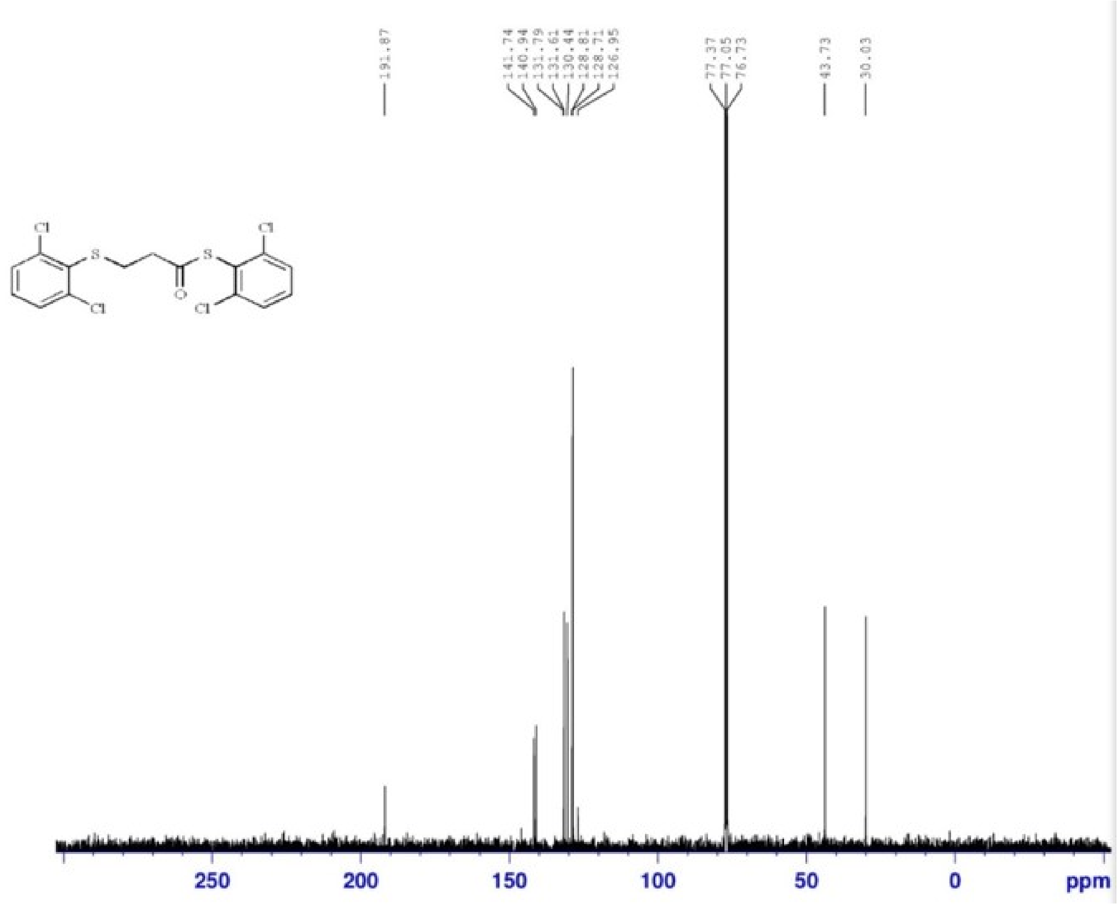
191.87
141.74
140.94
131.79
131.61
130.44
128.81
128.71
126.95
77.37
77.05
76.73
43.73
30.03
Cl
S
O
250
200
150
100
50
0
ppm

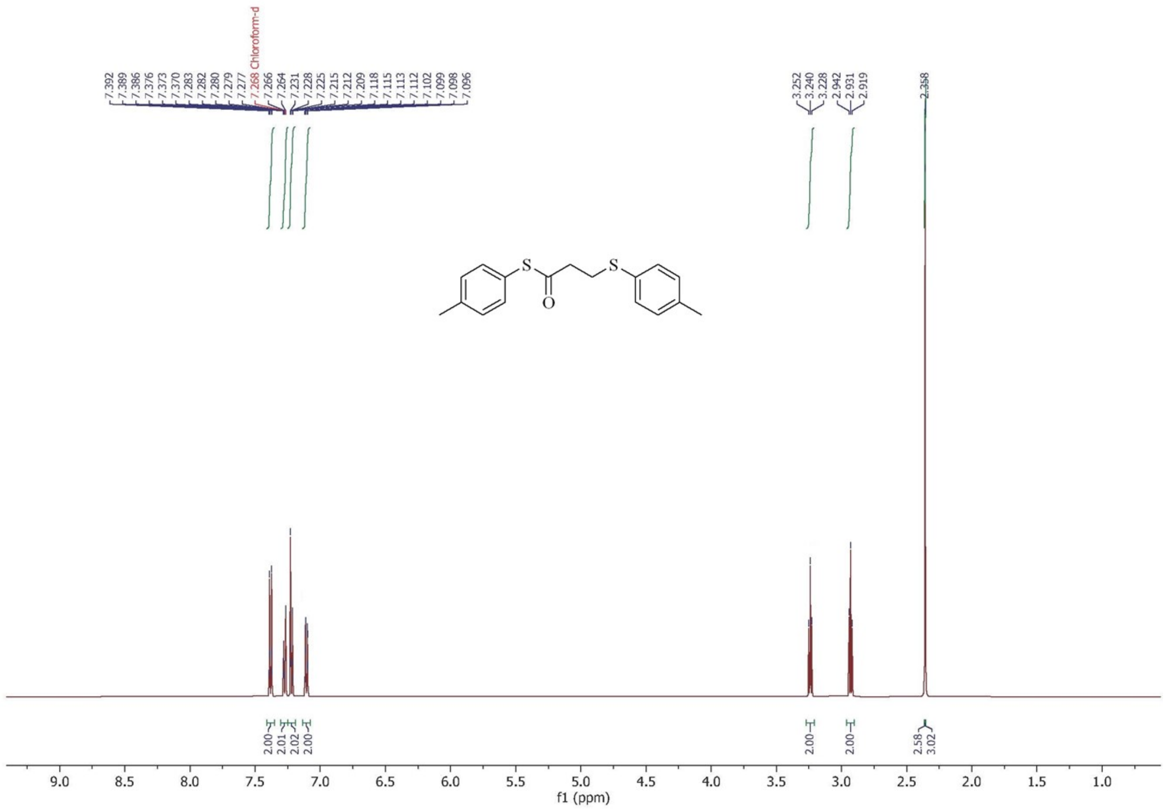
7.392
7.389
7.386
7.376
7.373
7.370
7.283
7.282
7.280
7.279
7.277
7.268 Chloroform-d
7.266
7.264
7.231
7.228
7.225
7.215
7.212
7.209
7.118
7.115
7.113
7.112
7.102
7.099
7.098
7.096
3.252
3.240
3.228
2.942
2.931
2.919
2.358
S
S
O
2.00
2.01
2.02
2.00
2.00
2.00
2.58
3.02
9.0 8.5 8.0 7.5 7.0 6.5 6.0 5.5 5.0 4.5 4.0 3.5 3.0 2.5 2.0 1.5 1.0
f1 (ppm)

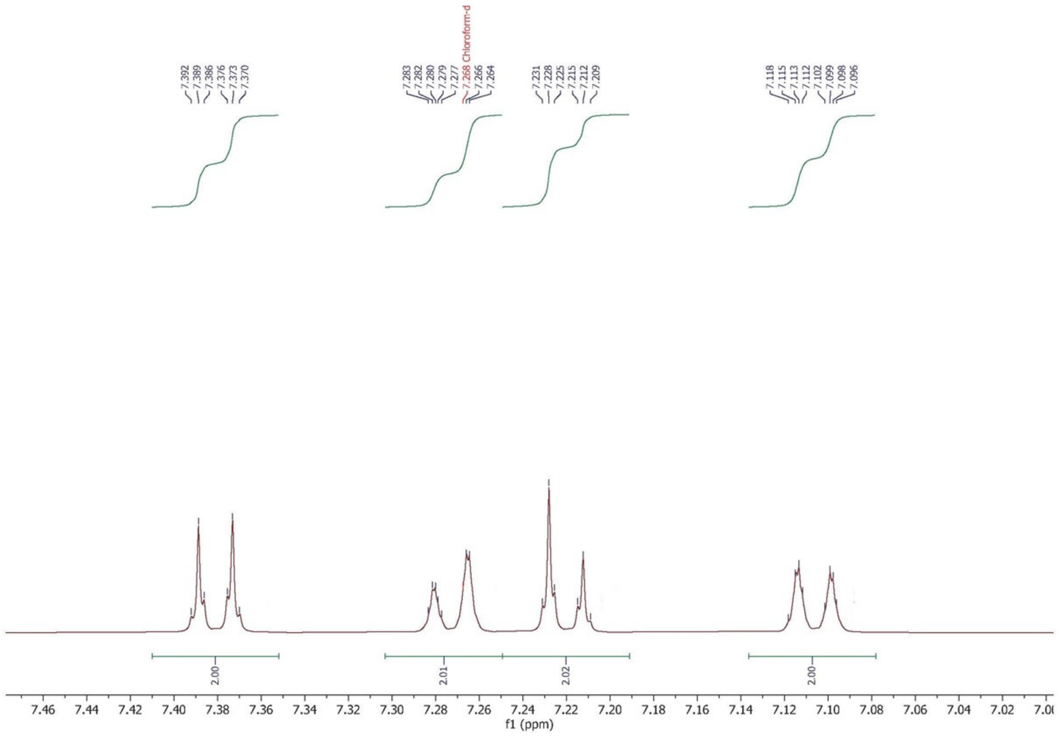
7.392
7.389
7.386
7.376
7.373
7.370
7.283
7.282
7.280
7.279
7.277
7.268 Chloroform-d
7.266
7.264
7.231
7.228
7.225
7.215
7.212
7.209
7.118
7.115
7.113
7.112
7.102
7.099
7.098
7.096
2.00
2.01
2.02
2.00
7.46 7.44 7.42 7.40 7.38 7.36 7.34 7.32 7.30 7.28 7.26 7.24 7.22 7.20 7.18 7.16 7.14 7.12 7.10 7.08 7.06 7.04 7.02 7.0
f1 (ppm)

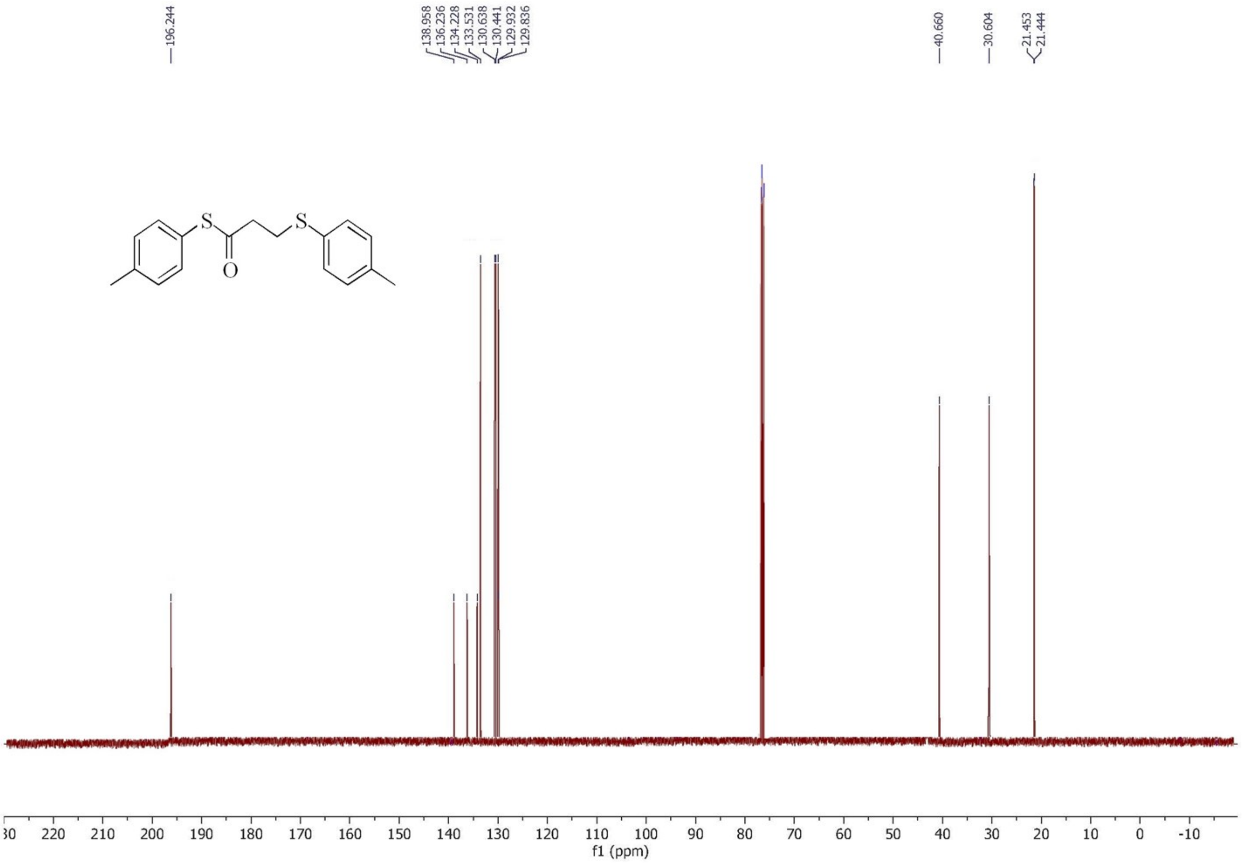

196.244
138.958
136.236
134.228
133.531
130.638
130.441
129.932
129.836
40.660
30.604
21.453
21.444
S
S
O
30 220 210 200 190 180 170 160 150 140 130 120 110 100 90 80 70 60 50 40 30 20 10 0 -10
f1 (ppm)

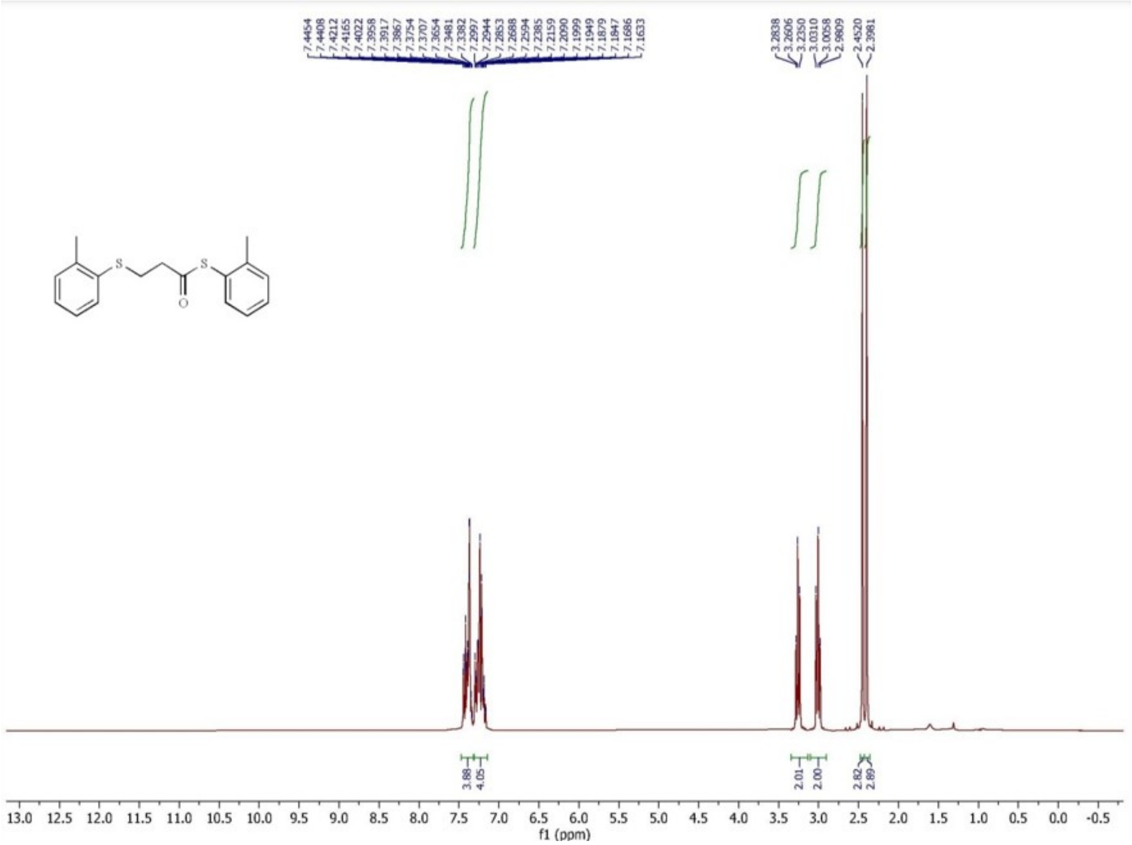

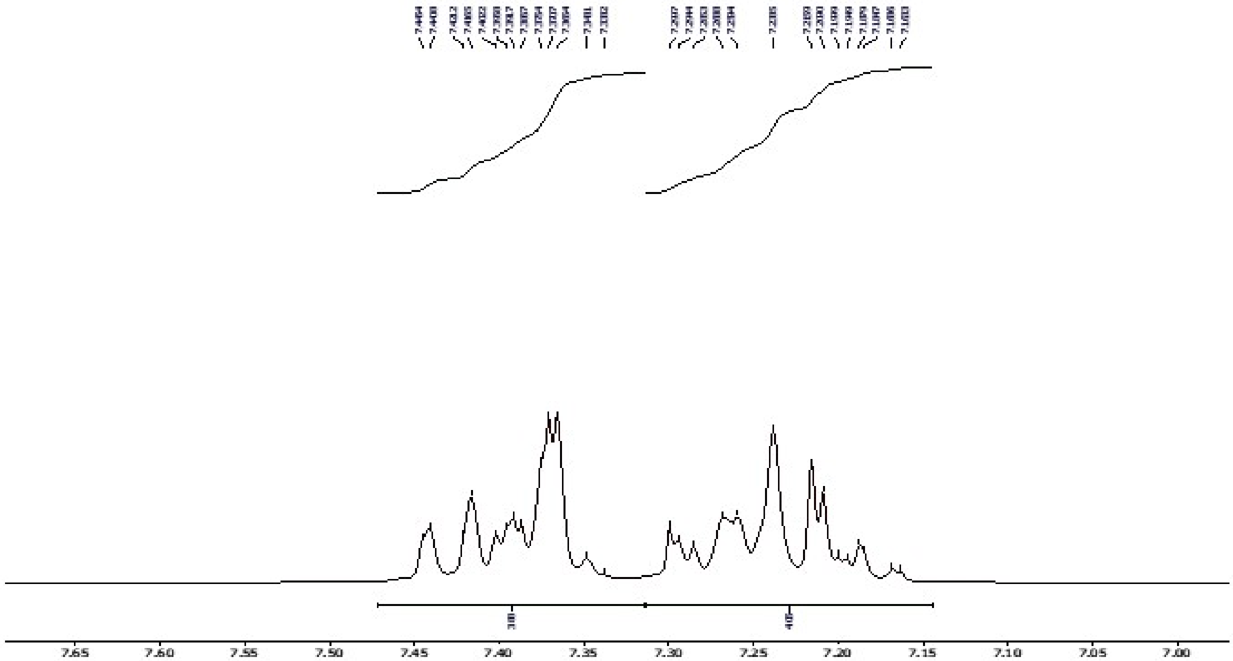

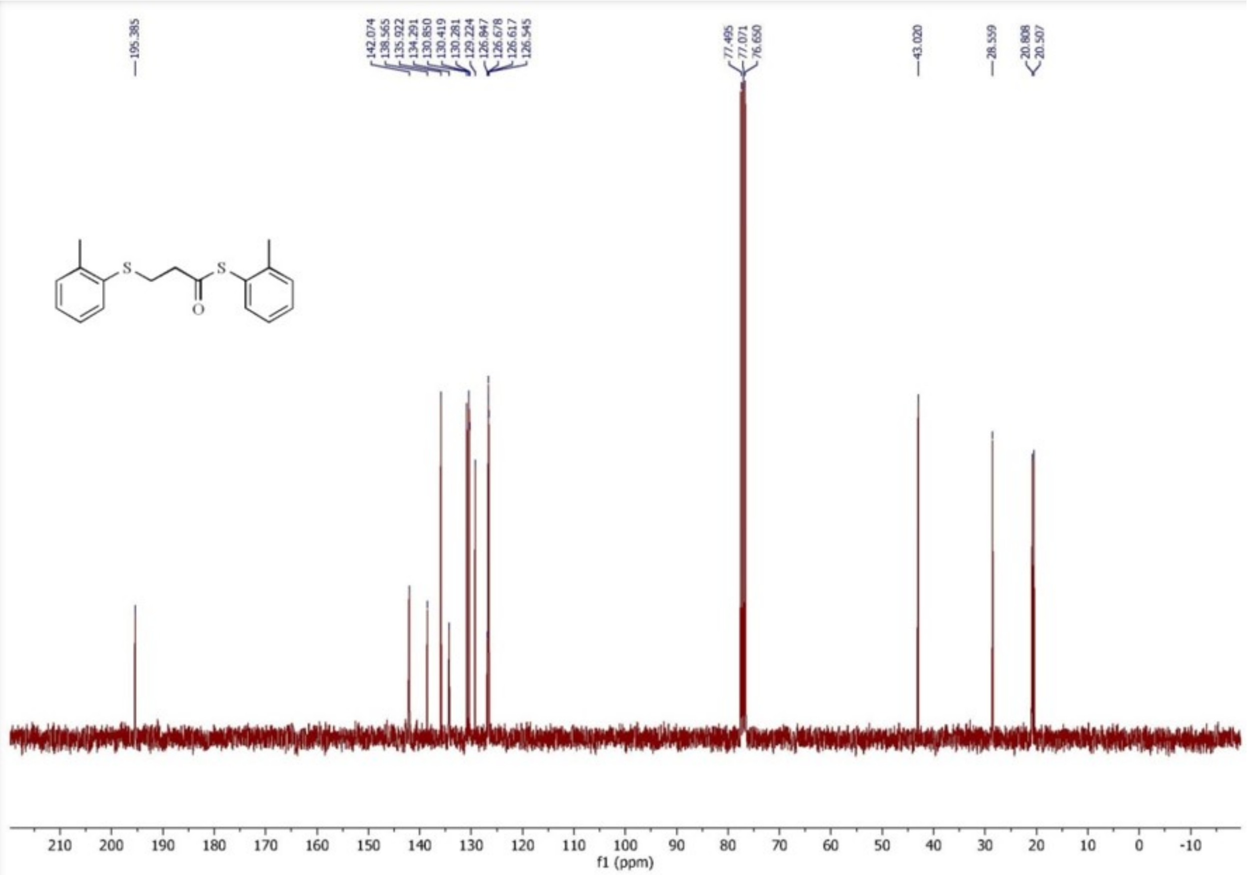

195.385
142.074
138.565
135.922
134.291
130.850
130.419
130.281
129.224
126.847
126.678
126.617
126.545
77.495
77.071
76.650
43.020
28.559
20.808
20.507
S
S
O
210 200 190 180 170 160 150 140 130 120 110 100 90 80 70 60 50 40 30 20 10 0 -10
f1 (ppm)

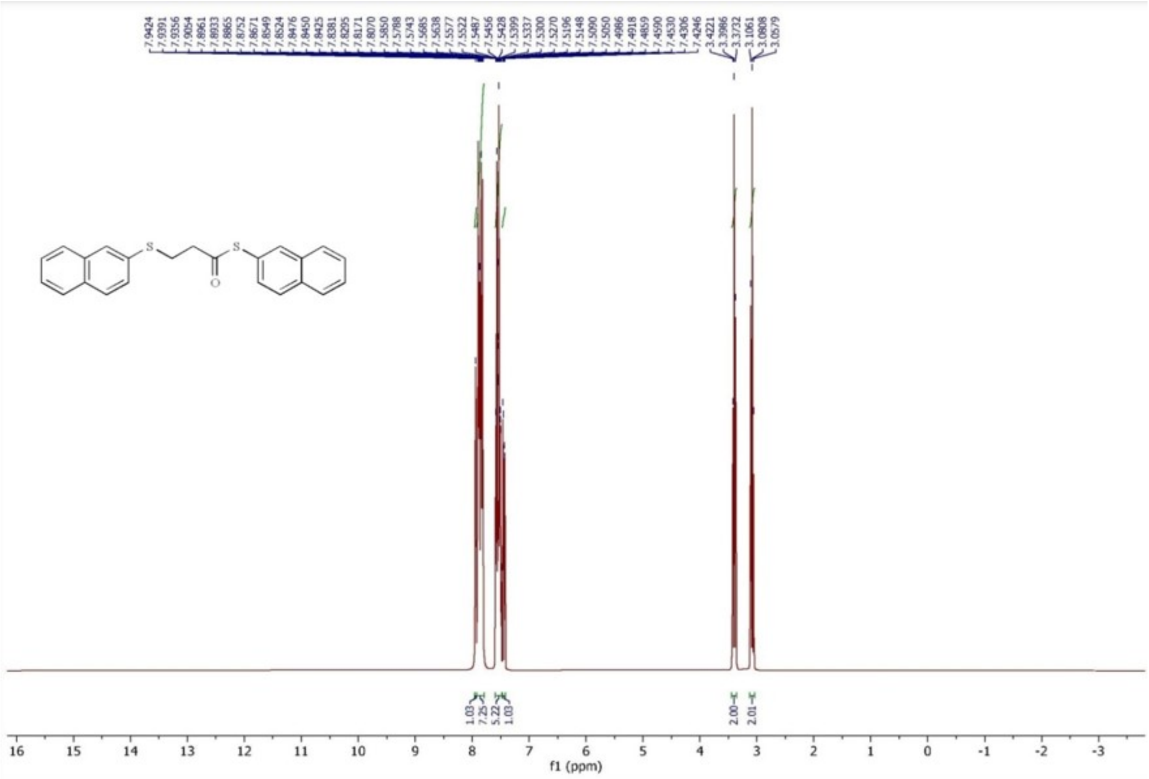
7.9424
7.9391
7.9356
7.9054
7.8961
7.8933
7.8865
7.8752
7.8671
7.8549
7.8524
7.8476
7.8450
7.8425
7.8381
7.8295
7.8171
7.8070
7.5850
7.5788
7.5743
7.5685
7.5638
7.5577
7.5522
7.5487
7.5456
7.5428
7.5399
7.5337
7.5300
7.5270
7.5196
7.5148
7.5090
7.5050
7.4986
7.4918
7.4859
7.4590
7.4530
7.4306
7.4246
3.4221
3.3986
3.3732
3.1061
3.0808
3.0579
S
S
O
1.03
7.25
5.22
1.03
2.00
2.01
16 15 14 13 12 11 10 9 8 7 6 5 4 3 2 1 0 -1 -2 -3
f1 (ppm)

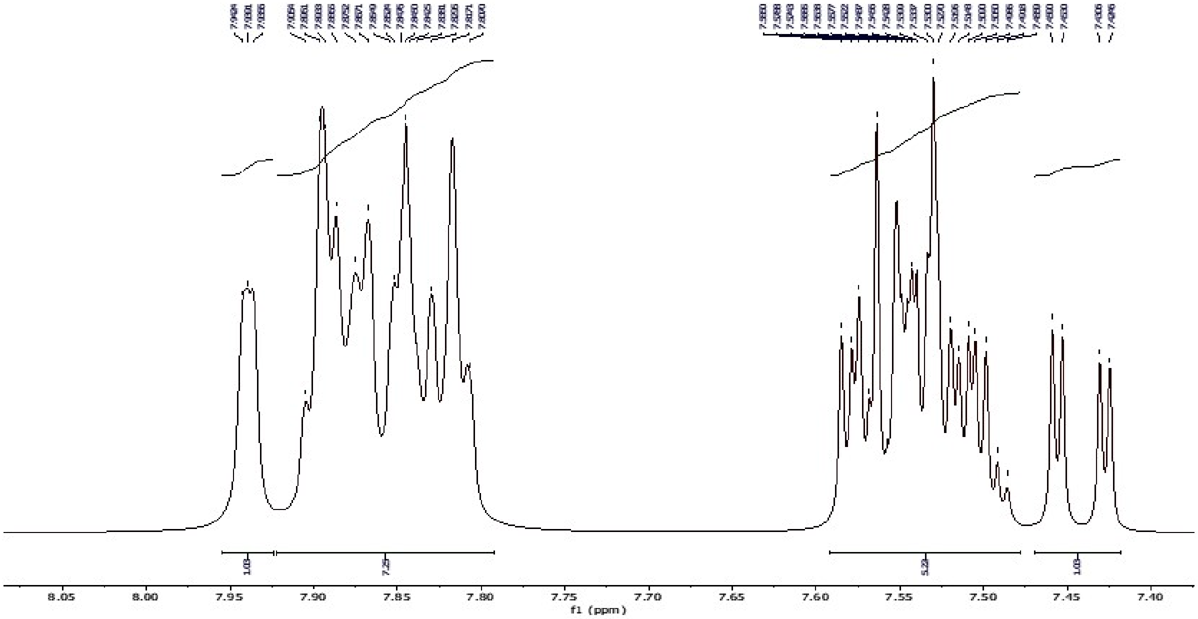
1.03
7.25
5.22
1.03
8.05 8.00 7.95 7.90 7.85 7.80 7.75 7.70 7.65 7.60 7.55 7.50 7.45 7.40
f1 (ppm)

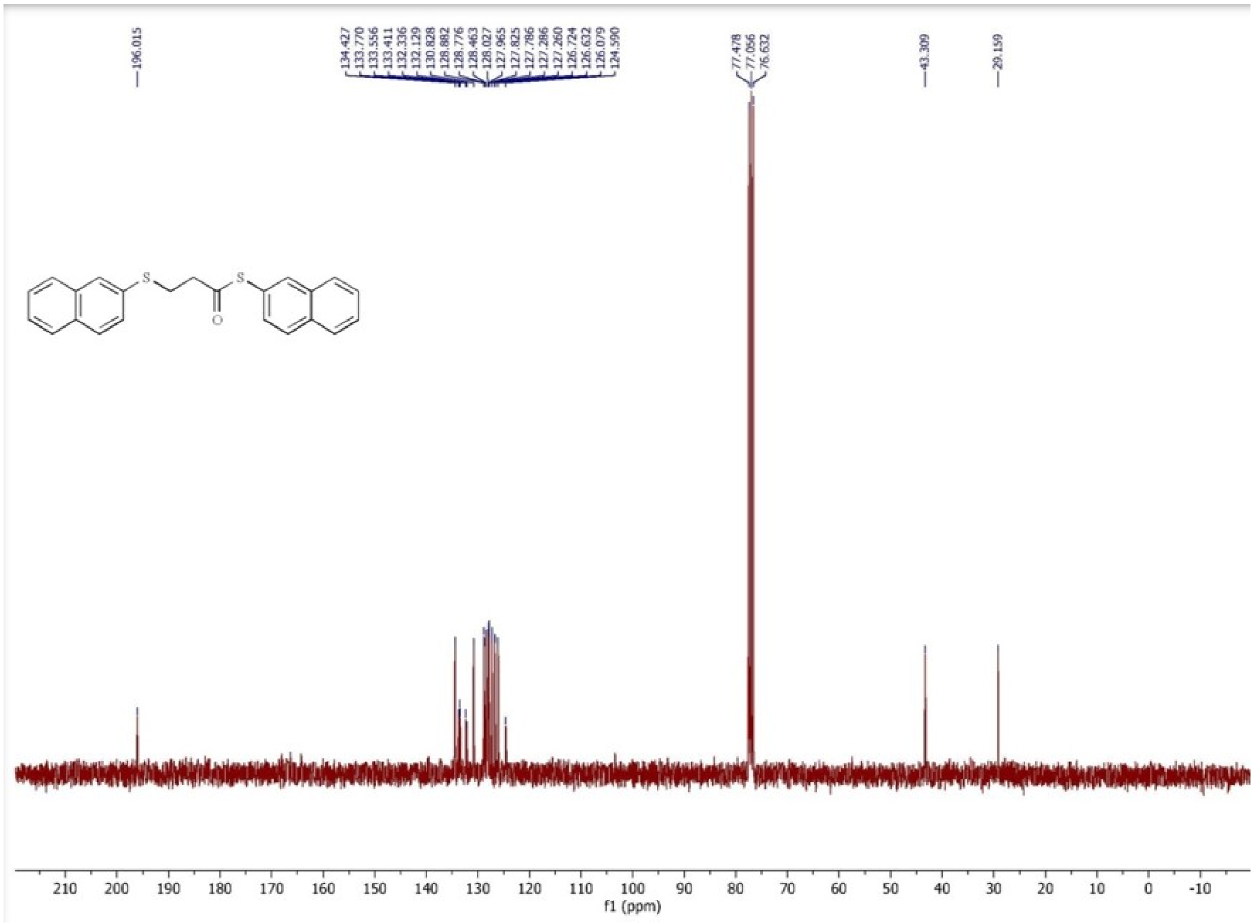
196.015
134.427
133.770
133.556
133.411
132.336
132.129
130.828
128.882
128.776
128.463
128.027
127.965
127.825
127.786
127.286
127.260
126.724
126.632
126.079
124.590
77.478
77.056
76.632
43.309
29.159
S
S
O
210 200 190 180 170 160 150 140 130 120 110 100 90 80 70 60 50 40 30 20 10 0 -10
f1 (ppm)
